# Heterogeneity in association of myocardial injury and mortality in sepsis or acute respiratory distress syndrome by subphenotype: a retrospective study

**DOI:** 10.1186/s13054-025-05613-2

**Published:** 2025-08-19

**Authors:** Pablo Amador Sanchez, Sarah Obeidalla, V. Eric Kerchberger, Andrew R. Moore, Manoj V. Maddali, Kirsten N. Kangelaris, Carolyn M. Hendrickson, Bruno Evrard, Kathleen D. Liu, Julie A. Bastarache, Michael A. Matthay, Angela J. Rogers, Carolyn S. Calfee

**Affiliations:** 1 Division of Cardiology, Department of Medicine, University of California San Francisco, San Francisco, CA USA; 2 Division of Allergy, Pulmonary, and Critical Care Medicine, Department of Medicine, Vanderbilt University Medical Center, Nashville, TN USA; 3 Division of Pulmonary, Allergy and Critical Care Medicine, Department of Medicine, Stanford University, Stanford, CA USA; 4 Division of Hospital Medicine, University of California San Francisco, San Francisco, CA USA; 5 Division of Allergy, Pulmonary, and Critical Care Medicine, Department of Medicine, Zuckerberg San Francisco General Hospital and Trauma Center, San Francisco, CA USA; 6 Inserm CIC 1435, Dupuytren Teaching Hospital, 87000 Limoges, France; 7 Division of Nephrology, Department of Medicine, University of California San Francisco, San Francisco, CA USA; 8 Cardiovascular Research Institute, University of California San Francisco, San Francisco, CA USA; 9 Department of Anesthesiology, University of California San Francisco, San Francisco, CA USA

## Abstract

**Rationale:**

Myocardial injury is common in acute respiratory distress syndrome (ARDS) and sepsis and associated with increased mortality. Two latent class analysis derived subphenotypes are associated with differential risk of mortality in these populations, though the association of troponin-I with mortality within each subphenotype is unknown.

**Methods:**

The derivation (n = 597 in EARLI) and validation (n = 452 in VALID) cohorts consisted of patients with sepsis or ARDS admitted to the ICU and enrolled in two separate prospective observational studies. Patients with troponin-I measured between hospital presentation and within 24 h of ICU admission were included. A parsimonious classifier model using interleukin-8, soluble tumor necrosis factor receptor-1, and vasopressor use assigned patients to subphenotype. Association between peak troponin-I concentration and 60-day in-hospital mortality within each subphenotype was assessed through logistic regression adjusting for age, admission laboratory values, vasopressor use, invasive ventilation use, and cardiac comorbidities.

**Results:**

Median peak troponin-I was significantly higher in the hyperinflammatory vs hypoinflammatory subphenotype in both cohorts (0.07 vs 0.04 ng/mL and 0.17 vs 0.07 ng/mL, both p < 0.05). The association between peak troponin-I and mortality differed between inflammatory subphenotypes (p-interaction 0.004, EARLI). In EARLI, each doubling of peak troponin-I was associated with increased adjusted odds of 60-day mortality (aOR 1.14, 95% CI 1.02–1.28) in the hypoinflammatory subphenotype only. These findings were corroborated in VALID (aOR 1.11, 95% CI 1.03–1.21 in hypoinflammatory).

**Conclusions:**

Admission peak troponin-I is significantly associated with 60-day mortality in patients with sepsis or ARDS. This association was distinctly driven by the hypoinflammatory subphenotype.

**Supplementary Information:**

The online version contains supplementary material available at 10.1186/s13054-025-05613-2.

## Introduction

Sepsis and the acute respiratory distress syndrome (ARDS) are clinical entities that reflect both an inciting insult and the host response to injury, and are characterized by significant heterogeneity in their patient population [1, 2]. This heterogeneity has made prognostication difficult and targeted pharmacotherapy equally elusive. In the last decade, advances combining complex statistical modelling alongside proteomic analysis have identified distinct subphenotypes in ARDS [3–7]. Across multiple randomized control trials and observational cohorts, latent class analysis (LCA) has revealed two molecular subphenotypes in ARDS, including one characterized by increased levels of inflammatory cytokines; thus, these are termed hyperinflammatory and hypoinflammatory. These subphenotypes not only differ in their clinical profile and outcomes but are also associated with remarkable heterogeneity in treatment response to therapies including steroids [8], statins [9], and diuretics [4]. More recently, these same subphenotypes were identified in broader populations of patients with sepsis [10] suggesting that this schema may be generally applicable to critical illness syndromes with inflammatory dysregulation.

Myocardial injury and troponin elevation during critical illness in sepsis and ARDS are common [11]. It may be related to increased oxygen demand, decreased oxygen supply, or direct myocardial injury. In ARDS, inherent pulmonary vascular dysfunction additionally introduces right ventricular stress as an agent of myocardial injury [12–16]. Early hospital troponin levels have been independently associated with worse mortality in both ARDS and sepsis [13, 14, 17], while other reports call into question the strength of this association, particularly after critical illness severity adjustment [12, 18–20]. One potential explanation for this variability is that prior studies evaluated patients with sepsis and ARDS without accounting for biological subphenotypes. The distinct inflammatory subphenotypes in both sepsis and ARDS introduce a novel lens to study the role of troponin in prognostication.

We sought to investigate whether myocardial injury is differentially associated with adjusted risk of mortality in each subphenotype among patients with sepsis or ARDS. The aims of this study were to (1) evaluate whether serum peak admission troponin-I differs by inflammatory subphenotype, and (2) evaluate the association between peak troponin-I and in-hospital mortality within each subphenotype.

## Methods

### Study design

This retrospective study analyzed data from two separate, prospectively enrolled, observational cohorts: Early Assessment of Renal and Lung Injury (EARLI) and Validating Acute Lung Injury markers for Diagnosis (VALID). The derivation cohort (EARLI) consisted of critically ill patients admitted to UCSF Medical Center and Zuckerberg San Francisco General Hospital. Briefly, patients were identified and enrolled in the emergency department (ED) once medical intensive care unit (ICU) admission was requested. The day of ICU admission was designated as day 1. The validation cohort (VALID) consisted of critically ill patients admitted to Vanderbilt University Medical Center. Patients were enrolled on the day after admission to a medical, surgical, trauma or cardiovascular ICU (day 2). To include similar patients between cohorts, we excluded VALID patients who had been admitted to the hospital more than 24 h prior to ICU admission, and patients admitted with trauma. Detailed study protocols for both cohorts have been described elsewhere [6, 21]. Adjudication of sepsis and ARDS was performed by a two-physician consensus, after thorough review of the clinical history and data; these physicians were blinded to biomarker data. ARDS and sepsis were defined by the American-European Consensus Conference (AECC) and Sepsis-2 criteria, respectively, to allow inclusion of patients enrolled prior to development of the Berlin definition and Sepsis-3 criteria [22–24]. Patients were included if they (1) had adjudicated sepsis or ARDS before day 2, (2) had complete clinical and biomarker data for classification through a parsimonious logistic regression model (PCM) [25], and (3) had clinically obtained serum troponin-I within 1 day of ICU admission. e-Fig. 1 shows the Consort diagram of patient selection. The primary clinical outcome was in-hospital mortality censored at 60-days. EARLI was approved by the UCSF institutional review board (IRB, #10-02852) and VALID by the Vanderbilt IRB (#051065). Patients or surrogates were consented for participation when possible, as previously described [6, 21].

### Data collection

Online supplemental methods details information regarding patient demographic and clinical data collection. Briefly, comprehensive data at the time of admission were captured from the electronic health record (EHR) by trained research coordinators. Clinically obtained troponin-I was extracted from the EHR. In EARLI, the peak troponin-I value between presentation to the ED through the first day of ICU admission, was captured. Because VALID enrolls patients admitted from the ED, the regular floor or hospital transfer, the peak troponin-I value within 1 day before ICU admission through the first day of ICU admission, was captured. Details on the assays are available in the supplemental methods.

### Biospecimen analysis

The concentrations of several plasma biomarkers known to be implicated in ARDS and sepsis biology were analyzed in EARLI (interleukin-6 = IL-6, interleukin-8 = IL-8, soluble tumor necrosis factor receptor-1 = sTNFr-1, intracellular adhesion molecule-1 = ICAM-1, plasminogen activator inhibitor-1 = PAI-1, protein-C, angiopoietin 2 = Ang-2), and in VALID (IL-8, and sTNFr-1). Plasma specimens were obtained either in the ED or ICU day 1 (EARLI) or on ICU day 2 (VALID). Biomarker assay procedural details are available in the supplemental methods. Several parsimonious classifier models (PCM), comprised of 3–4 serum biomarkers and clinical variables, were previously validated by our group to classify ARDS and sepsis patients with high fidelity compared to LCA [6, 25]. For both EARLI and VALID, a 3-variable PCM utilizing IL-8, sTNFr-1 and vasopressor use on ICU admission, was used for subphenotype assignment [25]. This PCM was chosen because its components had the least missingness in the EARLI dataset. As a sensitivity analysis, an alternate 3-variable model utilizing IL-8, sTNFr-1 and bicarbonate was used to evaluate the impact that the binary variable of vasopressor use had on patient classification and main results. A probability cutoff of ≥ 0.5 was used for assignment to hyperinflammatory subphenotype. More details on selection of the specific PCM are available in the supplemental methods.

### Statistical analysis

Continuous numerical variables were expressed as median (interquartile range) and were compared using Mann–Whitney U test or Kruskal–Wallis test, as appropriate. Categorical variables were expressed as frequencies and percentages and compared using the chi-square test. Descriptive statistics on demographics and clinical characteristics were stratified by subphenotype assignment. To evaluate patient differences by degree of myocardial injury, demographics and clinical characteristics were also stratified by tertiles of peak troponin-I. Clinical predictors of in-hospital mortality were assessed through univariable logistic regression (e-Table 1). We tested the interaction between peak troponin-I concentration and in-hospital mortality by dichotomous subphenotype assignment, through a logistic regression model using log_2_-transformed troponin-I, subphenotype, and their product as independent variables and in-hospital mortality as the dependent variable. The PCM generates probabilities of belonging to the hyperinflammatory subphenotype; we repeated the above procedure with continuous probability of belonging to the hypoinflammatory subphenotype.

Multivariable logistic regression was used to evaluate the effect size of peak troponin-I on in-hospital mortality in the overall cohort and within each subphenotype, while adjusting for important clinical covariates. Covariate selection was based on a directed acyclic graph based on knowledge of existing literature (e-Fig. 2) [26]. APACHE II score was disaggregated, and its relevant components were included as covariates in the models. We found no evidence of multicollinearity by variable inflation factor, among included covariates. The main results are reported among patients with clinically obtained admission troponin-I values. To assess the impact of missing troponin-I on regression analyses, we performed a sensitivity analysis after imputation, in EARLI. Troponin-I was assumed to be missing at random (MAR) as related to the value of troponin-I itself, but not randomly as related to variables of illness severity and patient comorbidities [27]. We performed 50 imputations with 50 iterations via chained equations, checking that convergence was achieved by visualizing mean and standard deviation of imputed troponin-I. The covariates included in the regression models were used as auxiliary variables. We used the *mice* package in R [28]. To determine the impact of diagnosis of sepsis or ARDS on the above models, sensitivity analyses were performed including (1) only patients with sepsis, and (2) only patients with ARDS. As an exploratory analysis, to evaluate whether changes in admission troponin-I were associated with mortality in either subphenotype, we modeled longitudinal troponin-I by the interaction of time and in-hospital mortality using linear mixed modeling. We adjusted these analyses by the confounders in e-Fig. 2.

To explore the relationship between available biomarkers and peak troponin-I, we constructed a Spearman correlation map of the patients in EARLI, as well as two separate Spearman networks of each inflammatory subphenotype. The networks were restricted to rho > 0.1, and adjusted p-values < 0.05 by the Benjamini–Hochberg procedure [29]. Reported *p*-values were two-sided, and significance assessed at ⍺-level of 0.05. Data was analyzed and visualized using R version 4.3.2 [30]. The Spearman correlation map was constructed using the package *ggraph*, and the Spearman correlation network was constructed using the R package *corrplot* [31, 32].

## Results

### Distinct characteristics across inflammatory subphenotypes

In EARLI, a total of 1679 patients were recruited between 2008 and 2021 and had complete data for clinical phenotyping at time of data pull. 597 of these patients met inclusion criteria and were selected for analysis (e-Fig. 1). As seen in Table 1, 571 patients (95.6%) were diagnosed with sepsis and 292 (54.1%) met AECC criteria for ARDS. The PCM assigned 394 patients (66.0%) to the hypoinflammatory subphenotype and 203 patients (34.0%) to the hyperinflammatory. As seen in e-Table 2, compared to the hypoinflammatory subphenotype, the hyperinflammatory had higher median concentrations of biomarkers related to inflammation (IL-6 and IL-8), leukocyte recruitment (ICAM-1), vascular permeability (Ang-2), and thrombosis (PAI-1). Biomarker and clinical characteristics in these two subphenotypes were distinct and consistent with the previously described inflammatory subphenotypes (e-Figs. 3 and 4) [25].

**Table 1:** Clinical characteristics, ICU therapies and outcomes stratified by inflammatory subphenotype in EARLI

	Overall *n* = *597*	Hypoinflammatory *n* = *394*	Hyperinflammatory *n* = *203*	*p*-value
Demographics				
Age, years	68 [57, 80]	67 [56, 81]	68 [58, 79]	1
Gender/sex (female), %	258 (43.2)	168 (42.6)	90 (44.3)	0.76
Race—Caucasian, %	272 (45.6)	190 (48.2)	82 (40.4)	0.08
BMI, kg/m^2^	24.7 [21.2, 29.2]	24.9 [21.1, 30.2]	24.2 [21.3, 27.2]	0.09
Comorbidities				
Hypertension, %	276 (46.2)	195 (49.5)	81 (39.9)	0.032
Diabetes, %	179 (30.0)	113 (28.7)	66 (32.5)	0.38
Coronary artery disease, %	112 (18.8)	77 (19.5)	35 (17.2)	0.57
Acute coronary syndrome, %	53 (8.9)	36 (9.1)	17 (8.4)	0.87
Congestive heart failure, %	159 (26.6)	110 (27.9)	49 (24.1)	0.37
Chronic kidney disease, %	111 (18.6)	75 (19.0)	36 (17.7)	0.78
COPD, %	122 (20.4)	95 (24.1)	27 (13.3)	0.003
Current smoker, %	97 (16.2)	66 (16.8)	31 (15.3)	0.73
Interstitial lung disease, %	13 (2.2)	10 (2.5)	3 (1.5)	0.59
Cirrhosis, %	39 (6.5)	19 (4.8)	20 (9.9)	0.029
Cardiac arrest*, %	80 (13.4)	42 (10.7)	38 (18.7)	0.009
ICU therapies on admission				
Vasopressors, %	316 (52.9)	147 (37.3)	169 (83.3)	< 0.001
Mechanical ventilation, %	315 (52.8)	182 (46.2)	133 (65.5)	< 0.001
ARDS by AECC, %	292 (54.1)	167 (46.8)	125 (68.3)	< 0.001
Primary ARDS risk factor				0.001
Sepsis, %	108 (37.0)	45 (26.9)	63 (50.4)	
Pneumonia, %	109 (37.3)	73 (43.7)	36 (28.8)	
Aspiration, %	46 (15.8)	29 (17.4)	17 (13.6)	
Other, %	29 (9.9)	20 (12.0)	9 (7.2)	
Sepsis present, %	571 (95.6)	378 (95.9)	193 (95.1)	0.78
APACHE II	27 [20, 35]	25 [18, 31]	36 [28, 40]	< 0.001
Outcomes				
Ventilator-free days, d	25.0 [0.0, 28.0]	26.0 [17.0, 28.0]	3.0 [0.0, 25.0]	< 0.001
60-day mortality, %	203 (34.0)	94 (23.9)	109 (53.7)	< 0.001

Data are frequencies (%) or median [Q1, Q3]

*ACS* acute coronary syndrome, *AECC* American European Consensus Conference, *APACHE II* Acute Physiology and Chronic Health Evaluation II, *BMI* body mass index, *ETOH* alcohol, *FiO2* fraction of inspired oxygen, *HCO3* bicarbonate, *ILD* interstitial lung disease

Statistical significance denoted by p < 0.05. * Diagnosed at time of ICU admission

### Comorbidities and clinical characteristics

The EARLI cohort had a substantial prevalence of comorbidities, including 26.6% with congestive heart failure (Table 1). However, there were no significant differences in prevalence of cardiac comorbidities between subphenotypes. As seen in e-Table 3, patients in the hyperinflammatory subphenotype had significantly higher admission heart rate and lower mean arterial pressures; however, there were no significant differences in hypoxia categories or SpO_2_/FiO_2_ ratios between subphenotypes. Compared to the hypoinflammatory subphenotype, the hyperinflammatory had lower admission hemoglobin, higher median creatinine, and lactate. Patients in the hyperinflammatory subphenotype had higher prevalence of vasopressor use (83.3 vs 37.3%, p < 0.001) and mechanical ventilation (65.5 vs 46.2%, p < 0.001) on admission, compared to the hypoinflammatory (Table 1). The hyperinflammatory subphenotype had higher prevalence of ARDS (68.3 vs 46.8%, p < 0.001) and higher median APACHE II scores (36 vs 25, p < 0.001).

Clinical outcomes differed between subphenotypes (Table 1). Median ventilator-free days at 28-days were significantly lower in the hyperinflammatory subphenotype (3 vs 26 days, p < 0.001). Overall, 60-day in-hospital mortality was 34.0%, substantially higher in the hyperinflammatory compared to the hypoinflammatory subphenotype (53.7 vs 23.9%, p < 0.001).

### Troponin concentration

**Fig. 1:**
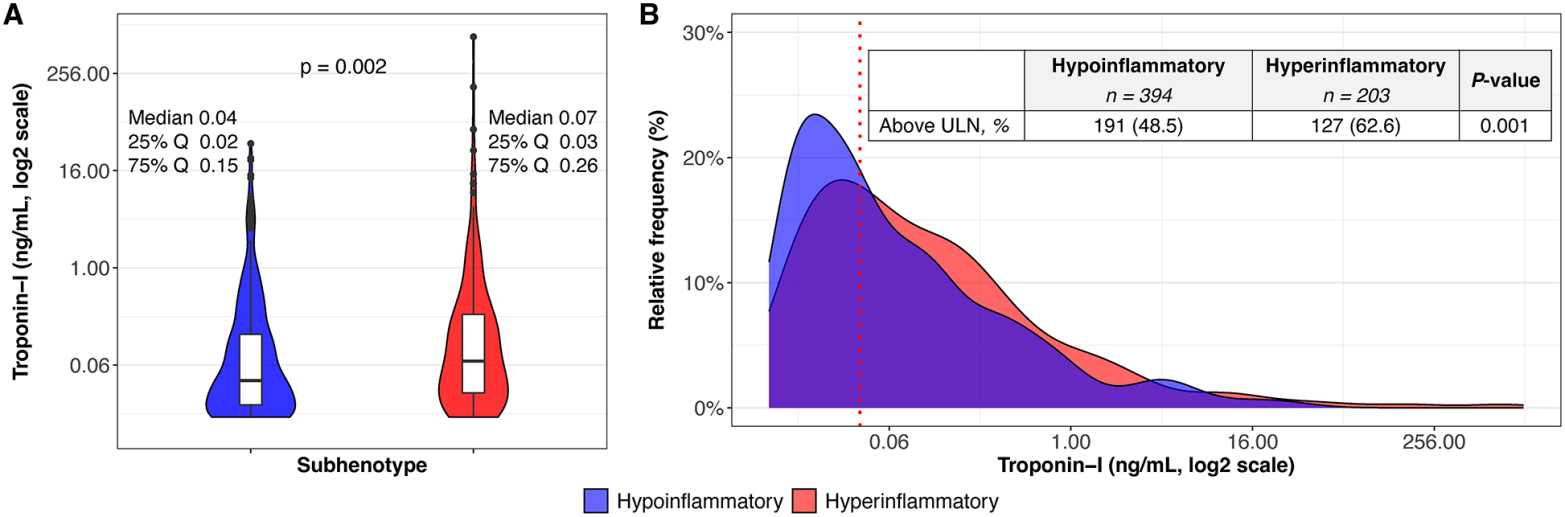
Median peak troponin-I higher in the hyperinflammatory compared to the hypoinflammatory (**A**). Higher proportion of elevated peak troponin-I above upper limit of normal for assay (ULN > 0.04ng/mL, red line, table insert) and a wider distribution of values in the hyperinflammatory phenotype (**B**)

As seen in Fig. 1, compared to the hypoinflammatory subphenotype, the hyperinflammatory had significantly higher median peak troponin-I (0.07 vs 0.04 ng/mL, p = 0.001, panel A), as well as a higher proportion of patients with concentrations above the upper limit of normal (62.6 vs 48.5%, p = 0.001, panel B). When comparing across tertiles of peak troponin-I, demographics were generally similar except for a trend of older age among higher tertiles (e-Table 4). Higher tertiles of peak troponin-I were also associated with significantly higher prevalence of coronary artery disease, congestive heart failure, and a diagnosis of acute coronary syndrome or cardiac arrest prior to ICU admission. Higher peak troponin-I tertiles tended to have higher white blood cell count and creatinine (e-Table 5). There were no differences in prevalence of vasopressor use, invasive mechanical ventilation, or diagnosis of ARDS by troponin-I tertile (e-Table 4).

### Association between troponin and mortality

**Fig. 2:**
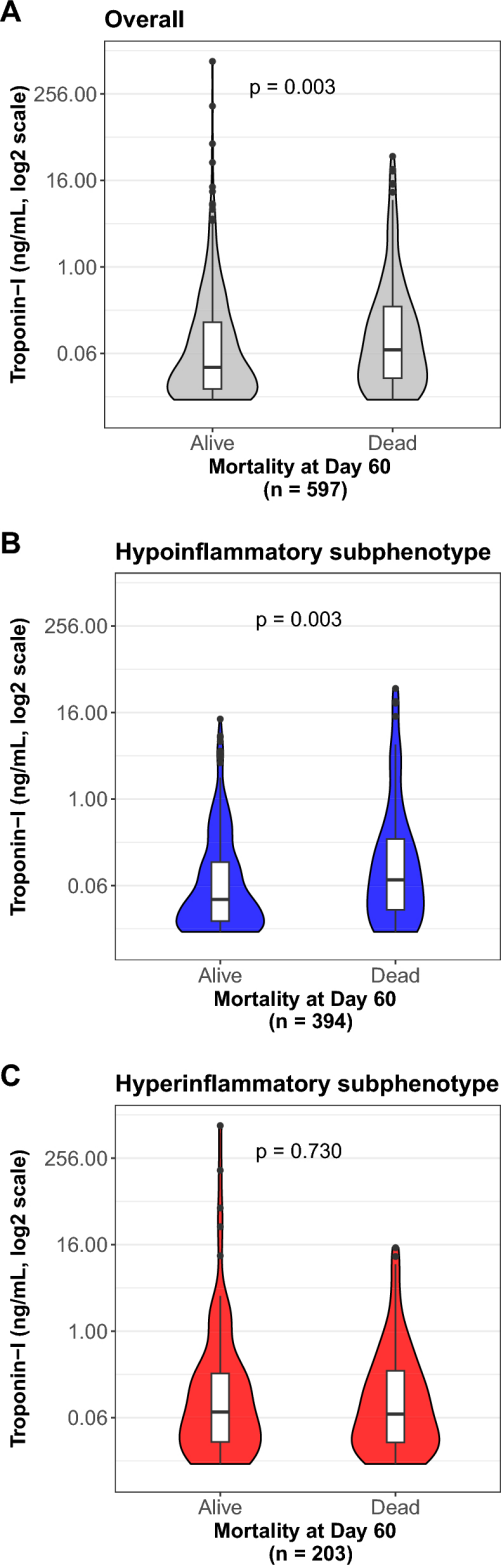
Median peak troponin-I by survival status at day 60. In the hypoinflammatory subphenotype, patients who died had higher peak troponin-I compared to those who survived (**B**); however, there were no differences in peak troponin-I by survival status within the hyperinflammatory subphenotype (**C**)

**Fig. 3:**
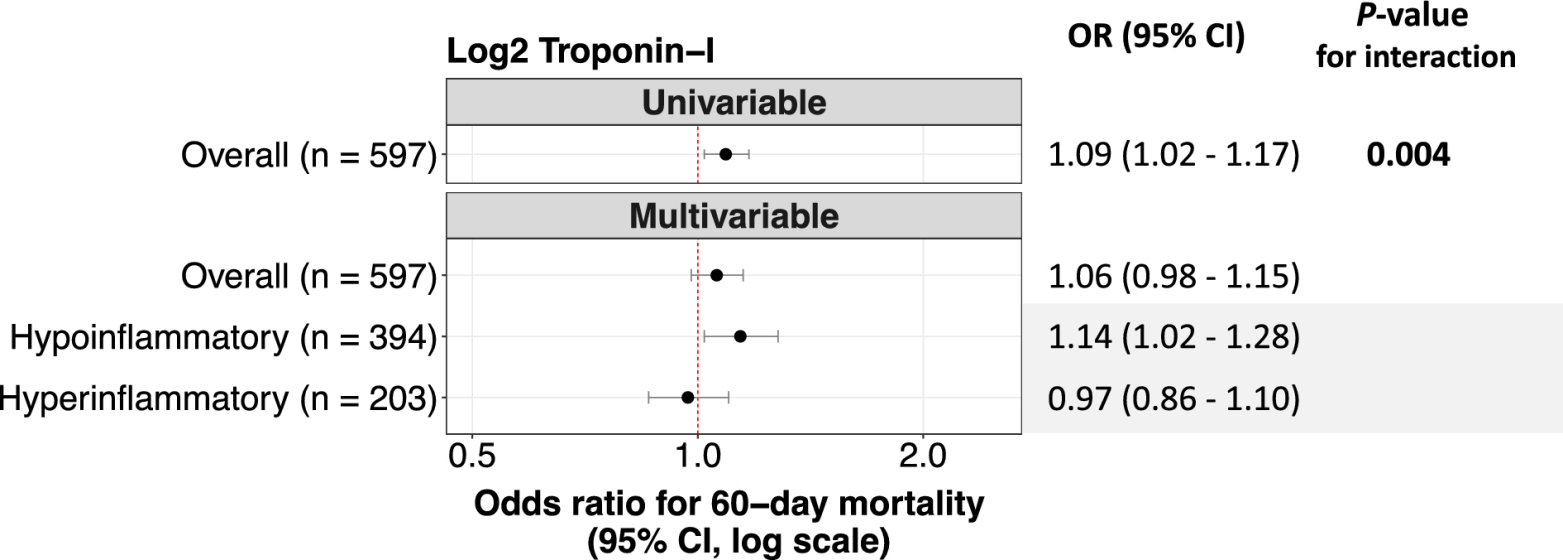
Forest plot with crude and adjusted odds ratios for 60-day mortality associated with peak troponin-I concentrations, in EARLI. Adjusted for age, hypertension, diabetes, coronary artery disease, congestive heart failure, acute coronary syndrome, admission hematocrit, log-transformed white blood cell count, log-transformed creatinine, respiratory rate, heart rate, vasopressor on day 1, and invasive ventilation on day 1

Four patients were still alive hospitalized past 60 days. As seen in Fig. 2A, compared to patients who survived, those who died had higher median peak troponin-I (0.07 vs 0.04 pg/mL, p = 0.002). In univariable analysis of the overall cohort, peak troponin-I was significantly associated with increased odds of in-hospital mortality (Fig. 3). The relationship between peak troponin-I and mortality differed between inflammatory subphenotypes (p-interaction = 0.004). Within the hypoinflammatory subphenotype, patients who died had higher peak troponin-I compared to those who survived (Fig. 2B); however, there was no difference by survival status within the hyperinflammatory subphenotype (Fig. 2C). As seen in Fig. 3**,** when inflammatory subphenotypes were evaluated in separate multivariable analyses, peak troponin-I was associated with a 1.14-fold increase in odds of in-hospital mortality per doubling of troponin-I concentration in the hypoinflammatory subphenotype (adjusted OR 1.14, 95% CI 1.02–1.28), while we found no association with mortality in the hyperinflammatory (adjusted OR 0.97, 95% CI 0.86–1.10). A sensitivity analysis using an alternate 3-variable PCM substituting bicarbonate for vasopressor use showed similar results (e-Fig. 5). Sensitivity analyses of (1) only patients with sepsis (n = 571) and (2) only patients with ARDS (n = 292), revealed the same heterogeneity in the association between peak troponin-I, inflammatory subphenotype, and mortality (e-Fig. 6).

**Fig. 4:**
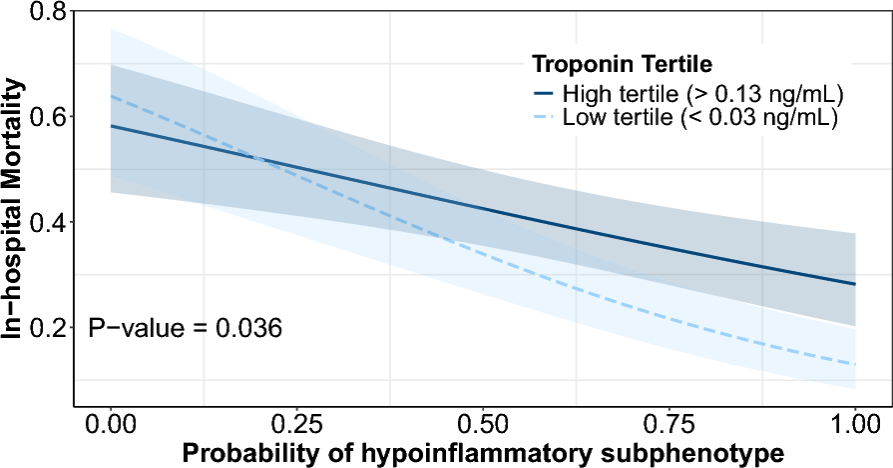
Differential probability of mortality according to probability of belonging to hypoinflammatory phenotype stratified by tertile of peak troponin-I concentration

The interaction between peak troponin-I and the continuous probability of subphenotype assignment provided by the PCM was also significant (p = 0.004). Figure 4 depicts the differential association between the probability of in-hospital mortality and probability of assignment to the hypoinflammatory subphenotype, stratified by highest and lowest tertile of peak troponin-I (p-interaction = 0.036). We observed an overall decreased probability of mortality as the probability of assignment to the hypoinflammatory subphenotype increased. However, this relationship was attenuated by troponin-I, where the highest troponin-I tertile was associated with higher mortality in the hypoinflammatory subphenotype only. In longitudinal analyses, as seen in e-Fig. 7A/B, in the hypoinflammatory subphenotype, in-hospital mortality was associated with an adjusted 1.56-fold (95% CI 1.12–2.16, p = 0.008) higher troponin-I concentration, though the rate of change was not different by survival status. In the hyperinflammatory subphenotype, troponin-I concentration was not associated with in-hospital mortality, though there was a non-significant adjusted 1.81-fold (95% CI 0.64–5.41, p for interaction = 0.27) faster daily rate of increase in troponin-I in patients who died (e-Fig. 7 A, B**)**.

### Biomarker relationships

Peak troponin-I had significant, albeit very weak, correlations with most biomarkers (e-Fig. 8). e-Figure 9 shows peak troponin-I had continued to have very weak significant correlations with biomarkers related to inflammation, pro-thrombosis and vascular integrity in the hypoinflammatory subphenotype only.

### External validation

From 2007–2019, VALID recruited a total of 2555 patients, and 452 met inclusion into our analysis. 452 patients (100%) had sepsis and 132 (29%) met AECC criteria for ARDS. The PCM assigned 359 (79.4%) as hypoinflammatory and 93 (20.6%) as hyperinflammatory. As in EARLI, the hyperinflammatory subphenotype had distinct biomarker profile, clinical characteristics and outcomes compared to the hypoinflammatory (e-Table 6). As seen in e-Table 7, median peak troponin-I was significantly higher for the hyperinflammatory compared to the hypoinflammatory subphenotype (0.17 vs 0.07, p = 0.021). The relationship between peak troponin-I and mortality again differed between subphenotypes in the same direction as in EARLI, though the p-value for interaction was not significant (p-interaction = 0.20). In multivariable analyses, after adjusting for the same covariates as the derivation cohort, each doubling of peak troponin-I was associated with a significant increase in odds of in-hospital mortality in the hypoinflammatory subphenotype (adjusted OR 1.11, 95% CI 1.03–1.21), while there was no association in the hyperinflammatory (e-Fig. 10).

### Sensitivity analysis after imputation of troponin-I

In EARLI, 273 of 870 patients (31.4%) did not have clinically obtained troponin-I on admission (e-Fig. 1). Compared to patients excluded because troponin-I was not obtained, those included were older, had more cardiac comorbidities, vasopressor and mechanical ventilation use, higher APACHE II and in-hospital mortality (e-Table 8). Patients without troponin-I had higher probability of assignment to the hypoinflammatory subphenotype compared to those with available troponin-I (76.9% vs 66.0%, p < 0.001). After imputation of peak troponin-I, the median troponin-I across 50 imputed datasets remained higher in the hyperinflammatory compared to the hypoinflammatory subphenotype (0.06 vs 0.04, p < 0.001). The results of the univariable and multivariable analyses did not change significantly after imputation (e-Fig. 11).

## Discussion

Our retrospective analysis of early myocardial injury in patients with sepsis or ARDS provided important insights. First, the hyperinflammatory subphenotype had significantly higher peak troponin-I compared to the hypoinflammatory. Second, there was a significant association between peak troponin-I and in-hospital mortality, but this association was distinctly driven by the hypoinflammatory subphenotype alone. Indeed, after multivariable adjustment, each doubling of peak troponin-I was associated with a 1.14-fold increase in odds of in-hospital mortality in the hypoinflammatory subphenotype only. Importantly, these findings were validated in a large independent cohort and sustained in multiple sensitivity analyses.

Myocardial injury during sepsis and ARDS is common. In both syndromes, the prevalence of elevated admission troponin is above 50%, and causes remain poorly understood [11, 12, 20]. Indeed, we had a similar prevalence in our cohorts. In the absence of Type 1 Myocardial Infarction [33], mechanisms of elevated troponin in sepsis may be related to increased oxygen demand (through tachycardia, adrenergic and metabolic tone), decreased oxygen supply (through hypoxemia, hypotension, or increased preload leading to decreased coronary perfusion pressure and myocyte stress), or direct myocardial injury (endotoxin, cytokine or free-radical-mediated injury, or pathogen-specific myocarditis). Mechanisms in ARDS somewhat mirror those in sepsis with the additional component of magnified right ventricular stress through pulmonary vascular dysfunction (right ventricular afterload) [12–15]. Importantly, prior publications have found the degree of troponin elevation as an independent predictor of mortality [13, 14, 17]. However, other reports found the association between troponin and mortality is significantly attenuated by adjustment for disease severity or presence of shock [12, 18–20]. This inconsistent association with mortality may be related to disease severity, timing of troponin assessment, or study population differences such as density of background comorbidities.

Some of the mechanisms plausibly associated with myocardial injury were more common in the hyperinflammatory subphenotype. That subphenotype had higher median heart rate, lower blood pressure, and more vasopressor use. Admission laboratory values of the hyperinflammatory subphenotype portrayed a sicker population, higher APACHE II score and a higher proportion diagnosed with ARDS. It is therefore not unexpected that the hyperinflammatory subphenotype, in both the derivation and validation cohorts, had higher admission troponin-I. However, the prevalence of pre-hospitalization cardiac comorbidities and acute coronary syndrome were not different between subphenotypes, suggesting that the higher troponin-I in the hyperinflammatory subphenotype may be due to factors related to acute critical illness. This finding is consistent with prior work from Sinha et al. evaluating the presence of LCA-based subphenotypes in COVID-19 ARDS where the hyperinflammatory subphenotype also had higher baseline troponin [8].

Interestingly, an association between peak troponin-I and mortality was only evident in the hypoinflammatory subphenotype. A pattern emerged when we evaluated the interaction between peak troponin-I concentration and the continuous probability of assignment to the hypoinflammatory subphenotype and their association with mortality (Fig. 4). Although the probability of mortality was lower among patients more likely to belong to the hypoinflammatory subphenotype, this relationship was attenuated significantly by the degree of troponin-I elevation, where patients with the highest tertile of troponin-I had a significantly higher mortality compared to the lowest tertile. This finding suggests a graduated value to peak troponin-I in its prediction of mortality, which diminished in patients that were least likely to belong to the hypoinflammatory subphenotype.

This heterogeneity may be due to several important factors. Because critically ill patients have synergistic drivers of mortality (e.g. multiple organ dysfunction), the predictive importance of peak troponin-I on outcomes is likely balanced by the magnitude of other insults accrued by the patient. It is possible that in the hyperinflammatory subphenotype due to higher rates of shock, organ dysfunction, and ARDS, peak troponin-I concentration does not discriminate outcomes. In contrast, in the less ill hypoinflammatory subphenotype, among a lower density of drivers of mortality, peak troponin-I provided valuable insight on the likelihood of mortality even after adjusting for baseline comorbidities and acute coronary syndrome.

Higher concentrations of inflammatory biomarkers, markers of vascular permeability, and pro-thrombosis have all been associated with worse outcomes in sepsis and ARDS [34–37]. The hyperinflammatory subphenotype is defined in part by significantly higher levels of these biomarkers compared to the hypoinflammatory, though there is a wide range and overlap in concentrations (e-Fig. 4A) [3, 6, 10]. Biomarker concentrations according to probabilities of subphenotype assignment suggest a gradient in inflammatory biology within and across subphenotypes (e-Fig. 4B). Notably, the patients for whom peak troponin-I was most associated with mortality had the highest probability of belonging to the hypoinflammatory subphenotype and the lowest concentrations of these biomarkers. Therefore, the association between peak troponin-I and mortality in these patients is less a direct result of acute inflammatory biology but significantly modulated by comorbidities. Conversely, exploratory analyses suggest the rate of rise in admission troponin-I may be differentially associated with in-hospital mortality in the hyperinflammatory subphenotype, in whom higher prevalence and severity of shock are expected. This is consistent with a recent report that death with severe underlying comorbidities was more common in the hypoinflammatory subphenotype of sepsis, while circulatory shock was the most common cause of death in the hyperinflammatory [38]. Larger, prospective studies, with protocolized collection and timing of troponin assays will be of paramount importance in evaluating this association.

Further characterization and risk stratification within each inflammatory subphenotype deserves attention. The hypoinflammatory subphenotype reliably comprises a majority of critically ill patients with sepsis or ARDS and though their risk of mortality is lower than the hyperinflammatory, there is residual risk heterogeneity for mortality within each subphenotype. Work from Moore et al. revealed that within the hypoinflammatory subphenotype, elevated serum IL-18 levels re-stratified patients into a higher risk category with significantly higher mortality [39]. These data emphasize both the potential for further enrichment in clinical trial populations as well as nuanced phenotyping. Our analyses similarly highlight peak admission troponin as an important biomarker for risk characterization in the hypoinflammatory subphenotype.

Whether there is an optimal timing of troponin measurement that can provide prognostic utility in both inflammatory subphenotypes remains unknown. A study of patients with severe sepsis and septic shock found that only admission troponin-T, and not an interval 3–6-h change, was independently associated with mortality [17]. Conversely, another study of patients with sepsis or septic shock found only the change in troponin-T from day 1 and day 2 to be predictive of mortality in the subset of patients with septic shock [40]. In an ARDS population, a study found that only progressive troponin-I elevation between day 1 and day 3 was an independent predictor of mortality in ARDS [13]. These studies suggest a variability in the importance of troponin for prediction of outcomes in the context of disease severity, timing of assay and trend. As our analysis showed, molecular subphenotyping enables identification of significant heterogeneity in these populations and a clearer picture of the value of troponin in outcome prognostication.

## Strength and limitations

Our study has notable strengths. The derivation and validation cohorts were large, and the prospective nature of both aids in mitigating bias. Determination of sepsis and ARDS was made by physician consensus. Concurrent assay of sepsis and ARDS-related biomarkers allowed a window into early admission pathophysiology. However, our study has important limitations. It is a retrospective analysis with inherent selection bias and confounding, which cannot be fully mitigated by adjustments. Because troponin-I was clinically obtained, 31.4% of patients in EARLI were excluded from the main analysis (e-Table 8). As explained, patients who did not have troponin-I available were less ill, had lower prevalence of cardiac comorbidities, and higher likelihood of assignment to the hypoinflammatory subphenotype, which suggests selection bias and limits generalizability. We hypothesized that troponin-I was not obtained in these patients at least in part due to the perceived patient illness severity and lack of symptoms implicating a cardiac complication. As such, imputation by available data to encapsulate clinical severity and comorbidities was a reasonable approach, limited by the variables captured in the dataset, though itself does not fully account for biases inherent to the study design [27]. This sensitivity analysis yielded similar results to the main findings (e-Fig. 9). Because patients in VALID are enrolled from the ED, floor or after transfer from outside hospital, troponin-I was less frequently obtained in that cohort. However, among patients in VALID admitted from the ED, 35% did not have clinically obtained troponin-I, like EARLI. The biospecimen collection day is consistently later by 1 day in VALID compared to EARLI. However, despite timing differences, prior publications note similar molecular classification across cohorts [6, 10]. Our main findings evaluate the effect size of peak troponin value on mortality while serial trends of troponin may be more informative depending on subphenotype [13]. We do not have granular data on previous or subsequent testing for degree of coronary artery disease, heart failure phenotype, or degree of baseline right or left ventricular dysfunction. These factors could impact both serum troponin concentration and its effect on mortality and represent residual confounding in our models. Our use of the Sepsis-2 and AECC ARDS definitions present a limitation in generalizability.

## Conclusions

In conclusion, clinically obtained peak troponin-I concentrations were higher among patients in the hyperinflammatory subphenotype of sepsis or ARDS, but only predictive of mortality the hypoinflammatory. These findings highlight peak troponin’s potential value in risk stratification within a subphenotype that deserves close attention. Subsequent studies should focus on protocolized assaying of troponin during hospitalization, evaluation of serial trends, their interaction with inflammatory subphenotype and association with clinical outcomes. Lastly, prospective multimodal evaluation of cardiac function and biochemical injury, their concordance, and whether mitigating them affects mortality in sepsis or ARDS populations will be consequential. Pending validation by prospective studies, future clinicians may tailor therapies by lowering cardiac demand, or closely monitoring cardiac injury, depending on troponin elevation on ICU admission. Clinical trials of such approaches would be needed.

## Supplementary Information

Supplementary Material 1.

## Data Availability

No datasets were generated or analysed during the current study.
