# Supplementary material for: Heterogeneity in association of myocardial injury and mortality in sepsis or acute respiratory distress syndrome by subphenotype: a retrospective study
Source: Crit Care. 2025 Aug 19;29:363. doi: 10.1186/s13054-025-05613-2 (PMC12363128; doi:10.1186/s13054-025-05613-2)
Supplement: Supplementary file 1 — Supplementary Material 1. [file 13054_2025_5613_MOESM1_ESM.pdf]

Heterogeneity in Association of Myocardial Injury and Mortality in Sepsis or Acute Respiratory Distress Syndrome by Subphenotype: A Retrospective Study.

Supplement

Pablo A. Sanchez[1], Sarah Obeidalla[2], V Eric Kerchberger[2], Andrew R. Moore[3], Manoj V. Maddali[3], Kirsten N. Kangelaris[4], Carolyn M. Hendrickson[5], Bruno Evrard[6], Kathleen D. Liu[7, 9], Julie A Bastarache[2], Michael A. Matthay[8,9], Angela J. Rogers[3], Carolyn S. Calfee[8,9].

1) Division of Cardiology, Department of Medicine, University of California San Francisco, San Francisco, CA, USA.

2) Division of Allergy, Pulmonary, and Critical Care Medicine, Department of Medicine, Vanderbilt University Medical Center, Nashville, Tennessee, USA.

3) Division of Pulmonary, Allergy and Critical Care Medicine, Department of Medicine, Stanford University, Stanford, CA, USA.

4) Division of Hospital Medicine, University of California San Francisco, San Francisco, CA, USA.

5) Division of Allergy, Pulmonary, and Critical Care Medicine, Department of Medicine, Zuckerberg San Francisco General Hospital and Trauma Center, San Francisco, CA, USA.

6) Inserm CIC 1435, Dupuytren Teaching Hospital, 87000, Limoges, France.

7) Division of Nephrology, Department of Medicine, University of California San Francisco, San Francisco, CA, USA.

8) Cardiovascular Research Institute, University of California San Francisco, San Francisco, CA, USA.

9) Department of Anesthesiology, University of California San Francisco, San Francisco, CA, USA.

Correspondence to: Pablo Amador Sanchez, MD. e-mail: pablo.sanchez@ucsf.edu

TABLE OF CONTENTS

Supplemental methods 3

Patient data collection 3

Troponin assay 3

Biospecimen procedure 3

Parsimonious classifier models 4

e-Tables 5

e-Table 1: Univariable logistic regression for 60-day mortality associated with clinical covariates in EARLI and VALID 5

e-Table 2: Selected biomarker concentrations stratified by inflammatory phenotype in EARLI 6

e-Table 3: Vital signs and laboratory data on admission stratified by inflammatory subphenotype, in EARLI 7

e-Table 4: Clinical characteristics of critically ill patients stratified by tertiles of peak troponin-I concentration in EARLI 9

e-Table 5: Vital signs and laboratory data on admission stratified by tertiles of peak troponin-I concentration in EARLI 11

e-Table 6: Clinical characteristics, ICU therapies, selected biomarkers and outcomes stratified by inflammatory subphenotype in VALID 13

e-Table 7: Peak troponin-I concentration stratified by inflammatory phenotype in VALID 15

e-Table 8: Comparison of clinical characteristics, ICU therapies and outcomes between patients included and those excluded because of missing admission troponin-I in EARLI 16

e-Figures 18

e-Figure 1: 18

e-Figure 2: 19

e-Figure 3: 20

e-Figure 4: 21

e-Figure 5: 22

e-Figure 6: 23

e-Figure 7: 24

e-Figure 8: 25

e-Figure 9: 26

e-Figure 10: 27

e-Figure 11: 28

e-References 29

SUPPLEMENTAL METHODS

Patient data collection

Comprehensive data on patient demographics, past medical history, and comorbidities at the time of admission were obtained through extraction from the electronic medical record or manually by trained research coordinators. Vital signs were captured at the time of enrollment, as were selected laboratory values performed as part of routine care. We calculated Acute Physiology and Chronic Health Evaluation II (APACHE II)[1] score at the time of enrollment. When available, PaO2/FiO2 ratio was used for determination of hypoxia and presence of ARDS, otherwise SpO2/FiO2 ratio was utilized.[2] Hypoxia category was converted to a 3-level categorical variable (mild 200 to < 300 mmHg, moderate 100 to < 200 mmHg, and severe < 100mmHg). Ventilator-free days were defined as follows: 1) 0 if the patient died within 28 days of first ICU admission, 2) 0 if the patient was mechanically ventilated for >= 28 days, in the first 28 days after first ICU admission, and 3) 28 - x, if patient was successfully liberated from ventilation < 28 days of first ICU admission, where x is cumulative ventilator days in the first 28 days.

Troponin assay

During the period of study enrollment, the UCSF hospital system used the Access AccuTnI assay (Beckman Coulter) for quantitation of cardiac troponin-I. The Vanderbilt University Medical Center used the Architect STAT Troponin-I (Abbott). The Access AccuTnI assay has a lower limit of detection of 0.01ng/mL, and a 99th percentile for normal of 0.04 ng/mL, where levels above this concentration are flagged as abnormal. The coefficient of variation at a level of 0.04 ng/mL is 10%.[3] The Architect STAT Troponin-I assay has a lower limit of detection of 0.009 ng/mL, and 99th percentile for normal of 0.028 ng/mL. The coefficient of variation at 0.032 ng/mL is 10%.[3] Values below the limit of quantitation were replaced by that value divided by the square root of 2 (n = 155 in EARLI, and n = 42 in VALID). Values above the upper limit of quantitation were replaced by the highest quantifiable value of the assay (n = 1 in EARLI and n = 1 in VALID).

Biospecimen procedure

Specimens were processed and stored at -80C, in small aliquots to minimize freeze-thaw cycles, until biomarker quantification. Singleplex assays were used for quantification of plasminogen activator inhibitor-1 (PAI-1, R&D systems, Minneapolis, MN) and protein C (Helena Laboratories, Beaumont, Tx). The rest of the biomarkers were run as single quantitation: soluble tumor necrosis factor receptor-1 (sTNFR-1), intercellular adhesion molecule-1 (ICAM-1), interleukin-6 (IL-6), interleukin-8 (IL-8), and angiopoietin-2 (Ang2) were measured using multiplex ELISA (R&D systems, Minneapolis, MN). Each analyzed plate followed a thorough quality control procedure. Protein biomarker levels below the limit of detection of the instrument were replaced by that value divided by the square root of 2 (IL-8 n = 4 in EARLI, sTNFR1 n = 4 in EARLI and n = 0 in VALID; ICAM-1 n = 5 in EARLI; Ang2 n = 1 in EARLI), and levels above the limit of detection were replaced by the highest quantifiable value for the assay (IL6 n = 15 in EARLI; IL-8 n = 7 in EARLI and n = 0 in VALID; sTNFR1 n = 7 in EARLI and n = 0 in VALID; ICAM-1 n = 6 in EARLI).

Parsimonious classifier models

Several parsimonious classifier models (PCM), comprised of 3-4 serum biomarkers and clinical variables, have been developed and validated to classify ARDS patients with high fidelity compared to LCA in randomized control trial and observational cohort populations.[4,5] All PCMs have similar performance.[5] Though derived and validated in ARDS populations, they have also been validated in sepsis populations.[6] For our study, the 3-variable model comprising IL-8, sTNFR-1 and vasopressor use was chosen, since its components had the least missingness in our dataset. IL-8 and sTNFR-1 concentrations were natural-log transformed prior to input into the PCM. The PCM provided the log odds of assignment to the hyperinflammatory phenotype, which were then converted to probabilities of phenotype assignment. A probability cutoff of > 0.5 was used for hyperinflammatory phenotype assignment, and the rest were assigned to the hypoinflammatory phenotype, as has been described previously.[5]

Parsimonious classifier models chosen:

Model Coefficients					
**Intercept**	**IL-8***	**sTNFR-1***	**Vasopressor**	**$HCO3^-$**	**AUC (95% CI)**
-18.4764	1.3013	1.3367	2.3439		0.94 (0.92-0.95)
-10.6110	1.2902	1.0732	--	-0.2326	0.95 (0.93-0.96)

IL = Interleukin, sTNFR-1 = soluble tumor necrosis factor receptor-1.* Coefficients were derived using natural log transformation of values for these variables. For IL-8 and sTNFR-1, a value of 1 was added to the measured value to allow log transformation.

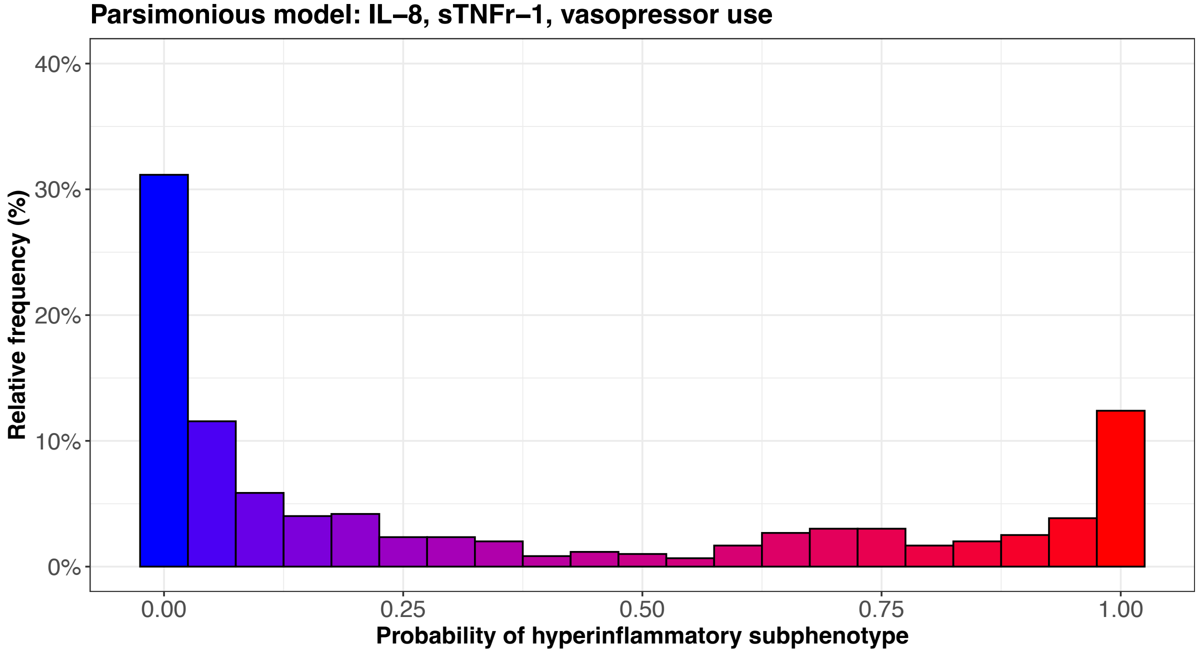

Histogram of probabilities generated by main PCM for assignment to the hyperinflammatory subphenotype.

e-Table 1: Univariable logistic regression for 60-day mortality associated with clinical covariates in EARLI and VALID.

60-day mortality

Univariable logistic regression

	**EARLI** *n = 597*		**VALID** *n = 452*	
	**OR**	**95% CI**	**OR**	**95% CI**
Age	1.02	1.01 - 1.04 *	1.03	1.01-1.04 *
Hypertension	0.74	0.53 - 1.04	1.20	0.81-1.92
Diabetes	0.97	0.67 - 1.40	1.05	0.69-1.60
Coronary artery disease	0.76	0.48 - 1.18	0.99	0.64-1.54
Acute coronary syndrome	0.83	0.44 - 1.50	0.61	0.24-1.55
Congestive heart failure	1.40	0.96 - 2.03	1.70	1.02-2.82*
Ln Creatinine	1.21	0.96 - 1.54	1.55	1.17-2.04 *
Heart rate	1.01	1.00 - 1.02 *	1.00	0.99-1.01
Respiratory rate	1.01	0.99 - 1.03	1.00	0.99-1.10
Hematocrit	0.96	0.94 - 0.98 *	0.96	0.99-0.99 *
Ln white blood cell count	0.80	0.64 – 1.00	0.82	0.58-1.2
Vasopressor on day 1	2.75	1.93 - 3.95 *	1.80	1.19-2.72 *
Invasive ventilation on day 1	2.69	1.89 - 3.86 *	1.07	0.70-1.62
APACHE II	1.12	1.09 - 1.14 *	1.06	1.04-1.09 *
Class: Hyperinflammatory	3.70	2.56 - 5.26 *	2.61	1.62-4.20 *
**Log_2 troponin-I**	1.09	1.02 - 1.17 *	1.10	1.04-1.17 *

APACHE II; Acute Physiology and Chronic Health Evaluation II; COPD, chronic obstructive pulmonary disease. * denotes $p < 0.05$.

e-Table 2: Selected biomarker concentrations stratified by inflammatory phenotype in EARLI.

	**Overall**<br>*n = 597*	**Hypoinflammatory**<br>*n = 394*	**Hyperinflammatory**<br>*n = 203*	***P*-value**
IL-6*, pg/mL*	102 [24, 718]	43 [16, 138]	1204 [205, 6539]	<0.001
IL-8*, pg/mL*	20 [9, 89]	12 [6, 21]	211 [59, 1267]	<0.001
TNFR-1*, pg/mL*	4226 [2060, 9545]	2796 [1556, 5060]	10436 [6426, 15602]	<0.001
ICAM-1*, pg/mL*	611036 [353823, 1049059]	503980 [327239, 849181]	809314 [511415, 1629036]	<0.001
Protein C,*% control*	76 [44, 115]	85 [52, 124]	60 [26, 92]	<0.001
PAI-1*, ng /mL*	7.0 [3.1, 22.1]	4.8 [2.6, 11.0]	23.1 [6.7, 51.8]	<0.001
Ang-2*, pg/mL*	6400 [3336, 12126]	4781 [2605, 8497]	12204 [6340, 20389]	<0.001

Data are median [Q1, Q3]. Compared to the hypoinflammatory class, the hyperinflammatory class was associated with significantly higher levels of every disease-related serum biomarker. Ang-2, angiopoietin 2; ICAM, intracellular adhesion molecule-1; IL, interleukin; PAI-1, plasminogen activator inhibitor-1; TNFR-1, soluble tumor necrosis factor receptor-1. Statistical significance denoted by $p < 0.05$.
Available biomarkers: Angiopoietin-2 (n = 596), ICAM-1 (n = 593), IL-6 (n = 593), IL-8 (n = 597), PAI1 (n = 592), protein-C (n = 591), sTNFR-1 (n = 597).

e-Table 3: Vital signs and laboratory data on admission stratified by inflammatory subphenotype, in EARLI.

	**Overall** *n = 597*	**Hypoinflammatory** *n = 394*	**Hyperinflammatory** *n = 203*	***P*-value**
Vitals on admission				
Temperature, *C*	37.5 [37.0, 38.3]	37.5 [37.0, 38.2]	37.6 [36.8, 38.5]	0.97
Heart rate, *bpm*	112 [97, 128]	108 [95, 125]	120 [103, 132]	<0.001
SBP, *mmHg*	86 [75, 103]	90 [79, 107]	79 [70, 89]	<0.001
MAP, *mmHg*	58 [51, 65]	60 [54, 68]	54 [46, 59]	<0.001
O2 saturation, *%*	92 [86, 95]	92 [87, 95]	92 [83, 96]	0.37
Hypoxia category				0.52
Mild, *%*	153 (38.8)	104 (40.5)	49 (35.8)	
Moderate, *%*	100 (25.4)	61 (23.7)	39 (28.5)	
Severe, *%*	141 (35.8)	92 (35.8)	49 (35.8)	
Respiratory rate	34 [29, 40]	33 [27, 40]	35 [30, 38]	0.16
Minute ventilation	10 [8, 13]	9 [8, 12]	11 [9, 14]	0.003
SpO2/FiO2 ratio	254 [97, 342]	257 [97, 392]	215 [95, 331]	0.41
**Labs on admission**				
White blood cell, *10^3 cells/ uL*	12.6 [7.9, 18.2]	13.05 [8.6, 18.4]	11.0 [5.4, 17.9]	<0.001
Hemoglobin, *g/dL*	11.2 [9.4, 13.2]	11.4 [9.5, 13.5]	11.0 [9.4, 12.8]	0.046
Hematocrit, *%*	34 [29, 40]	35 [29, 41]	34 [29, 39]	0.06
Platelets, *10^3 cells/ uL*	193 [132, 273]	214 [157, 286]	148 [91, 229]	<0.001
Sodium, *mmol/L*	136 [133, 140]	136 [133, 140]	136 [132, 139]	0.23
HCO3, *mEq/L*	22 [18, 26]	23 [20, 27]	19 [16, 23]	<0.001
BUN, *mg/dL*	29 [17, 49]	25 [15, 43]	37 [24, 58]	<0.001
Creatinine, *mg/dL*	1.40 [0.92, 2.35]	1.20 [0.84, 1.79]	2.14 [1.27, 3.32]	<0.001
Glucose, *mg/dL*	140 [112, 199]	142 [117, 194]	137 [100, 208]	0.07

Lactate, *mmol/L*	3.6 [2.5, 5.4]	3.2 [2.1, 4.5]	5.0 [3.7, 7.5]	<0.001
Albumin, *g/dL*	2.7 [2.2, 3.2]	2.9 [2.4, 3.4]	2.3 [1.8, 2.8]	<0.001
Total bilirubin, *mg/dL*	0.9 [0.6, 1.4]	0.8 [0.6, 1.2]	1.2 [0.7, 1.9]	<0.001
PaO2, *mmHg*	118 [76, 201]	107 [74, 192]	127 [80, 203]	0.19
PaCO2, *mmHg*	37 [31, 45]	41 [33, 51]	34 [29, 43]	<0.001

Data are frequencies (%) or median [Q1, Q3]. AECC, American European Consensus Conference; APACHE II, Acute Physiology and Chronic Health Evaluation II; BMI, body mass index; BUN, blood urea nitrogen; FiO2, fraction of inspired oxygen; HCO3, bicarbonate; MAP, mean arterial pressure; PaCO2, arterial partial pressure of carbon dioxide; PaO2, arterial partial pressure of oxygen; O2, oxygen; SBP, systolic blood pressure; SpO2, systemic oxygen saturation.

Vitals availability: Temperature (n = 597), heart rate (n = 597), SBP (n = 592), MAP (n = 597), O2 saturation (n = 592), hypoxia category (n = 394), respiratory rate (n = 597), minute ventilation (n = 196), SpO2/FiO2 ratio (n = 230).

Lab availability: WBC (n = 597), hemoglobin (n = 595), hematocrit (n = 597), platelets (n = 593), sodium (n = 597), bicarbonate (n = 577), creatinine (n = 597), BUN (n = 595), glucose (n = 591), lactate (n = 105), albumin (n = 313), total bilirubin (n = 392), PaO2 (n = 251), PaCO2 (n = 250).

e-Table 4: Clinical characteristics of critically ill patients stratified by tertiles of troponin-I concentration in EARLI.

	**Low tertile <0.03** *n = 199*	**Mid tertile 0.03 - 0.13** *n = 199*	**High tertile >0.13** *n = 199*	***P*-value**
Demographics				
Age, *years*	63 [52, 75]	73 [62, 83]	67 [57, 78]	<0.001
Gender/sex (female), *%*	86 (43.2)	84 (42.2)	88 (44.2)	0.92
Race – Caucasian, %	97 (48.7)	90 (45.2)	85 (42.7)	0.48
BMI, *kg/m2*	24 [21, 29]	24 [21, 29]	25 [22, 31]	0.18
Comorbidities				
Hypertension, *%*	86 (43.2)	102 (51.3)	88 (44.2)	0.22
Diabetes, *%*	55 (27.6)	63 (31.7)	61 (30.7)	0.66
Coronary artery disease, *%*	21 (10.6)	50 (25.1)	41 (20.6)	0.001
Acute coronary syndrome *%*	3 (1.5)	12 (6.0)	38 (19.1)	<0.001
Congestive heart failure, *%*	30 (15.1)	59 (29.6)	70 (35.2)	<0.001
Chronic kidney disease, *%*	31 (15.6)	42 (21.1)	38 (19.1)	0.34
COPD, *%*	43 (21.6)	45 (22.6)	34 (17.1)	0.35
Current smoker, *%*	44 (22.1)	22 (11.1)	31 (15.6)	0.011
Interstitial lung disease, *%*	4 (2.0)	4 (2.0)	5 (2.5)	0.92
Cirrhosis, *%*	13 (6.5)	12 (6.0)	14 (7.0)	0.92
Cardiac arrest, *%*	15 (7.5)	27 (13.6)	38 (19.1)	0.003
**ICU therapies on admission**				
Vasopressors, *%*	98 (49.2)	103 (51.8)	115 (57.8)	0.21
Invasive ventilation, *%*	105 (52.8)	97 (48.7)	113 (56.8)	0.28
ARDS by AECC, %	94 (53.1)	90 (49.5)	108 (59.7)	0.14
APACHE II	25 [18, 33]	28 [20, 35]	29 [22, 37]	<0.001

Data are frequencies (%) or median [Q1, Q3]. AECC, American European Consensus Conference; APACHE II, Acute Physiology and Chronic Health Evaluation II; ARDS, acute respiratory distress syndrome; BMI, body mass index; COPD, chronic obstructive pulmonary disease. Statistical significance denoted by $p < 0.05$.

e-Table 5. Vital signs and laboratory data on admission stratified by tertiles of troponin-I concentration in EARLI.

	**Low tertile** **<0.03** *n = 199*	**Mid tertile** **0.03 - 0.13** *n = 199*	**High tertile** **>0.13** *n = 199*	***P*-value**
Vitals on admission				
Temperature, *C*	37.5 [37.0, 38.3]	37.4 [37.0, 38.3]	37.6 [37.0, 38.4]	0.71
Heart rate, *beats/min*	110 [98, 124]	113 [95, 128]	112 [98, 130]	0.26
SBP, *mmHg*	88 [75, 105]	85 [76, 103]	85 [75, 99]	0.28
MAP, *mmHg*	58 [51, 65]	57 [51, 64]	58 [51, 65]	0.66
O2 saturation, *%*	92 [87, 95]	92 [85, 95]	91 [85, 96]	0.75
Hypoxia category				0.58
Mild, *%*	52 (43.0)	55 (40.4)	46 (33.6)	
Moderate, *%*	30 (24.8)	34 (25.0)	36 (26.3)	
Severe, *%*	39 (32.2)	47 (34.6)	55 (40.1)	
Respiratory rate, *breath/min*	34 [28, 40]	34 [29, 40]	34 [29, 39]	0.96
Minute ventilation, L/*min*	9 [8, 12]	10 [8, 13]	11 [8, 14]	0.09
SpO2/FiO2 ratio	257 [97, 414]	285 [100, 424]	213 [95, 314]	0.14
**Labs on admission**				
White blood cell, *10^3 cells/ uL*	10.7 [7.2, 16.1]	12.6 [8.6, 18.4]	13.5 [8.5, 19.3]	0.003
Hemoglobin, *g/dL*	11.7 [9.8, 13.6]	10.9 [9.3, 12.8]	11.1 [9.1, 13.2]	0.07
Hematocrit, *%*	36 [30, 42]	34 [29, 40]	34 [28, 40]	0.10
Platelets, *10^3 cells/ uL*	202 [142, 279]	209 [141, 294]	175 [110, 252]	0.002
Sodium, *mmol/L*	136 [133, 139]	136 [132, 139]	137 [133, 141]	0.015
HCO3, *mEq/L*	22 [19, 26]	22 [18, 25]	21 [17, 25]	0.19
BUN, *mg/dL*	21 [13, 39]	31 [20, 53]	31 [20, 54]	<0.001
Creatinine, *mg/dL*	1.07 [0.77, 1.93]	1.48 [1.06, 2.40]	1.66 [1.06, 2.86]	<0.001
Glucose, *mg/dL*	135 [111, 174]	138 [111, 201]	151 [115, 222]	0.035
Lactate, *mmol/L*	4.8 [2.5, 6.6]	3.9 [3.0, 5.0]	3.1 [2.1, 4.9]	0.27

Albumin, *g/dL*	2.8 [2.3, 3.4]	2.3 [1.9, 3.0]	2.6 [2.2, 3.3]	0.003
Total bilirubin, *mg/dL*	0.8 [0.5, 1.3]	0.8 [0.6, 1.5]	1.0 [0.6, 1.5]	0.09
PaO2, *mmHg*	103 [69, 173]	111 [77, 196]	127 [82, 212]	0.28
PaCO2, *mmHg*	38 [30, 47]	38 [32, 47]	36 [31, 44]	0.43

Data are frequencies (%) or median [Q1, Q3]. BUN, blood urea nitrogen; FiO2, fraction of inspired oxygen; HCO3, bicarbonate; MAP, mean arterial pressure; PaCO2, arterial partial pressure of carbon dioxide; PaO2, arterial partial pressure of oxygen; O2, oxygen; SBP, systolic blood pressure; SpO2, systemic oxygen saturation. Statistical significance denoted by $p < 0.05$.
Available labs and vitals as in e-Table 3

e-Table 6. Clinical characteristics, ICU therapies, selected biomarkers and outcomes stratified by inflammatory subphenotype in VALID.

	**Overall** *n = 452*	**Hypoinflammatory** *n = 359*	**Hyperinflammatory** *n = 93*	***P*-value**
Demographics				
Age, *years*	60 (50, 70)	60 (50, 70)	59 (52, 68)	0.7
Gender/sex (female), *%*	203 (45%)	156 (43%)	47 (51%)	0.2
Race – Caucasian, %	380 (84%)	299 (83%)	81 (87%)	0.4
BMI, *kg/m2*	28 (24, 33)	28 (24, 34)	28 (24, 32)	0.9
Comorbidities				
Hypertension, *%*	285 (63%)	222 (62%)	63 (68%)	0.3
Diabetes, *%*	171 (38%)	139 (39%)	32 (34%)	0.4
Coronary artery disease, *%*	141 (31%)	116 (32%)	25 (27%)	0.3
Acute coronary syndrome, *%*	29 (6.4%)	27 (7.5%)	2 (2.2%)	0.060
Congestive heart failure, *%*	80 (18%)	68 (19%)	12 (13%)	0.2
Chronic kidney disease, *%*	114 (25%)	84 (23%)	30 (32%)	0.080
COPD, *%*	95 (21%)	80 (22%)	15 (16%)	0.2
Current smoker, *%*	126 (28%)	105 (29%)	21 (23%)	0.2
Interstitial lung disease, *%*	2 (0.4%)	2 (0.6%)	0 (0%)	>0.9
Cirrhosis, *%*	34 (7.5%)	21 (5.8%)	13 (14%)	0.008
**ICU therapies on admission**				
Vasopressors, *%*	224 (50%)	146 (41%)	78 (84%)	<0.001
Mechanical ventilation, *%*	276 (61%)	215 (60%)	61 (66%)	0.3
ARDS by AECC, *%*	132 (29%)	105 (29%)	27 (29%)	>0.9
Sepsis present, *%*	452 (100%)	359 (100%)	93 (100%)	1.0
APACHE II	28 (22, 34)	27 (21, 32)	33 (28, 39)	<0.001
**ARDS and sepsis biomarkers**				
IL-8, *pg/mL*	19 (8, 59)	13 (7, 25)	216 (101, 558)	<0.001

sTNFR-1, *pg/mL*	3,908 (2,105, 6,991)	3,296 (1,945, 5,466)	11,328 (5,467, 19,879)	<0.001
**Outcomes**				
Ventilator-free days, *d*	25.0 (22.5, 28.0)	26.0 (23.0, 28.0)	25.0 (22.0, 28.0)	0.2
60-day mortality, *%*	132 (29%)	89 (25%)	43 (46%)	<0.001

Data are frequencies (%) or median [Q1, Q3]. AECC, American European Consensus Conference; APACHE II, Acute Physiology and Chronic Health Evaluation II; ARDS, acute respiratory distress syndrome; BMI, body mass index; COPD, chronic obstructive pulmonary disease, IL-8, interleukin 8; sTNFR-1, soluble tumor necrosis factor receptor-1. Statistical significance denoted by $p < 0.05$.

e-Table 7. Peak troponin-I concentration stratified by inflammatory phenotype in VALID.

	**Overall** *n = 452*	**Hypoinflammatory** *n = 359*	**Hyperinflammatory** *n = 93*	***P*-value**
Troponin-I, *ng/mL*	0.09 [0.02, 0.64]	0.07 [0.02, 0.56]	0.17 [0.03, 1.00)	0.019

Data are frequencies (%), median [Q1, Q3]. Troponin-I upper reference limit 0.028 ng/mL.

e-Table 8: Comparison of clinical characteristics, ICU therapies and outcomes between patients included and those excluded because of missing admission troponin-I in EARLI.

	**Troponin-I available** *n = 597*	**Troponin-I missing** *n = 273*	***P*-value**
Demographics			
Age, *years*	68 [57, 80]	60 [49, 70]	<0.001
Gender/sex (female), *%*	258 (43.2)	123 (45.1)	0.67
Race – Caucasian, %	272 (45.6)	148 (54.2)	0.02
BMI, *kg/m2*	24.71 [21.18, 29.28]	25.35 [21.49, 29.85]	0.25
Comorbidities			
Hypertension, *%*	276 (46.2)	105 (38.5)	0.038
Diabetes, *%*	179 (30.0)	55 (20.1)	0.003
Coronary artery disease, *%*	112 (18.8)	20 (7.3)	<0.001
Acute coronary syndrome, *%*	53 (8.9)	1 (0.4)	<0.001
Congestive heart failure, *%*	159 (26.6)	19 (7.0)	<0.001
Chronic kidney disease, *%*	111 (18.6)	40 (14.7)	0.18
COPD, *%*	122 (20.4)	31 (11.4)	0.002
Current smoker, *%*	97 (16.2)	24 (8.8)	0.004
Interstitial lung disease, *%*	13 (2.2)	8 (2.9)	0.67
Cirrhosis, *%*	39 (6.5)	16 (5.9)	0.82
Cardiac arrest, *%*	80 (13.4)	6 (2.2)	<0.001
Inflammatory phenotype			0.002
Hypoinflammatory	394 (66.0)	210 (76.9)	
Hyperinflammatory	203 (34.0)	63 (23.1)	
**ICU therapies on admission**			
Vasopressors, *%*	316 (52.9)	99 (36.3)	<0.001
Mechanical ventilation, *%*	315 (52.8)	71 (26.0)	<0.001
ARDS by AECC, *%*	292 (54.1)	76 (31.1)	<0.001

Primary ARDS risk factor			0.12
Sepsis, %	108 (37.0)	34 (44.7)	
Pneumonia, %	109 (37.3)	30 (39.5)	
Aspiration, %	46 (15.8)	4 (5.3)	
Other, %	29 (9.9)	8 (10.5)	
Sepsis present, %	571 (95.6)	268 (98.2)	0.01
APACHE II	27.00 [20.00, 35.00]	21.00 [15.00, 26.00]	<0.001
**Outcomes**			
Ventilator-free days, *d*	25.00 [0.00, 28.00]	28.00 [25.00, 28.00]	<0.001
60-day mortality, %	203 (34.0)	38 (13.9)	<0.001

Data are frequencies (%) or median [Q1, Q3]. AECC, American European Consensus Conference; APACHE II, Acute Physiology and Chronic Health Evaluation II; BMI, body mass index; COPD, chronic obstructive pulmonary disease. Statistical significance denoted by $p < 0.05$.

SUPPLEMENTARY FIGURES

e-Figure 1: Consort diagram.

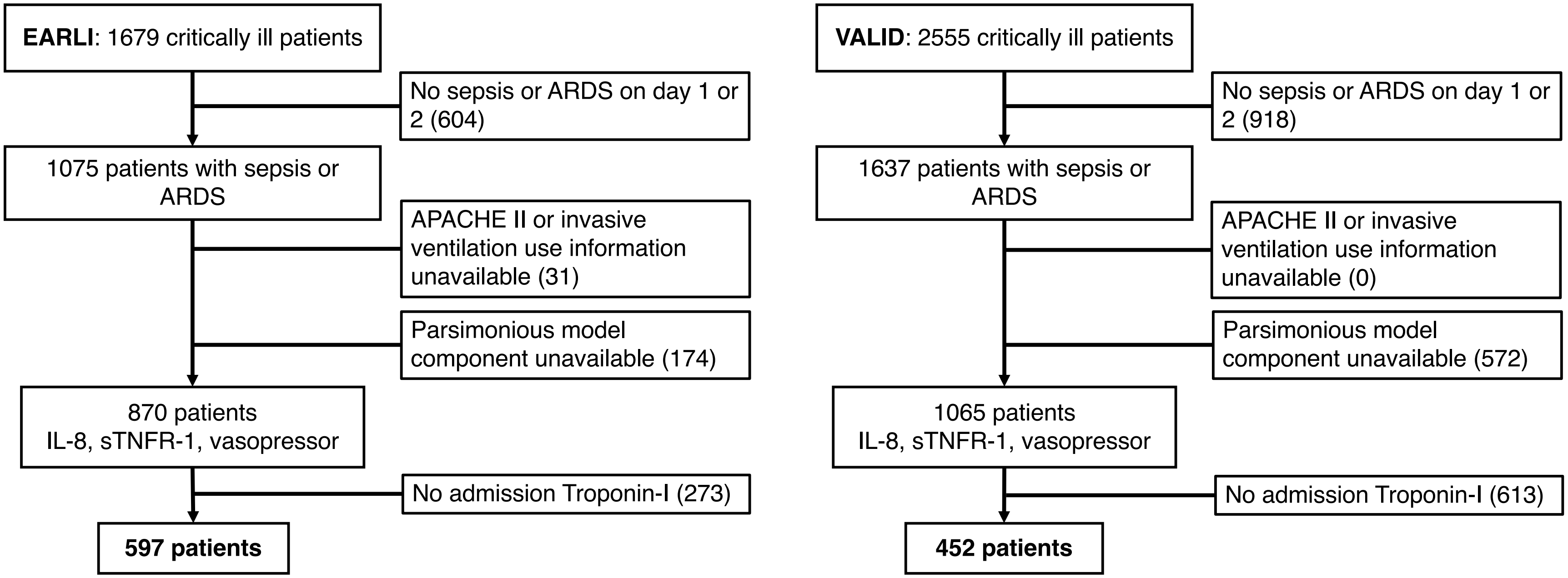

e-Figure 2: Directed Acyclic Graph (DAG) of selected variables used to identify potential confounders in the association between Troponin-I concentrations and 60-day mortality in EARLI.

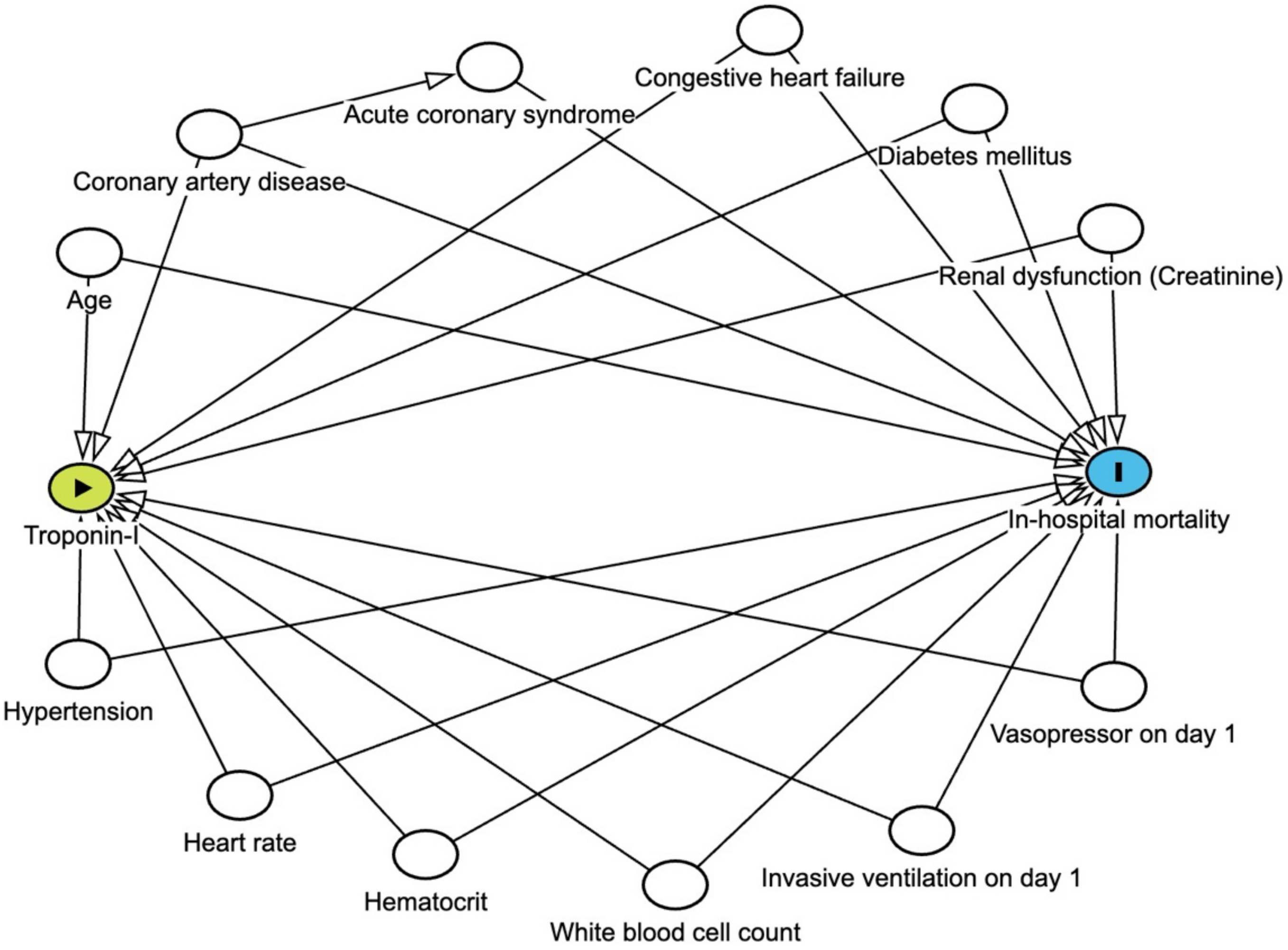

e-Figure 3: Standardized values for continuous selected variables in EARLI.

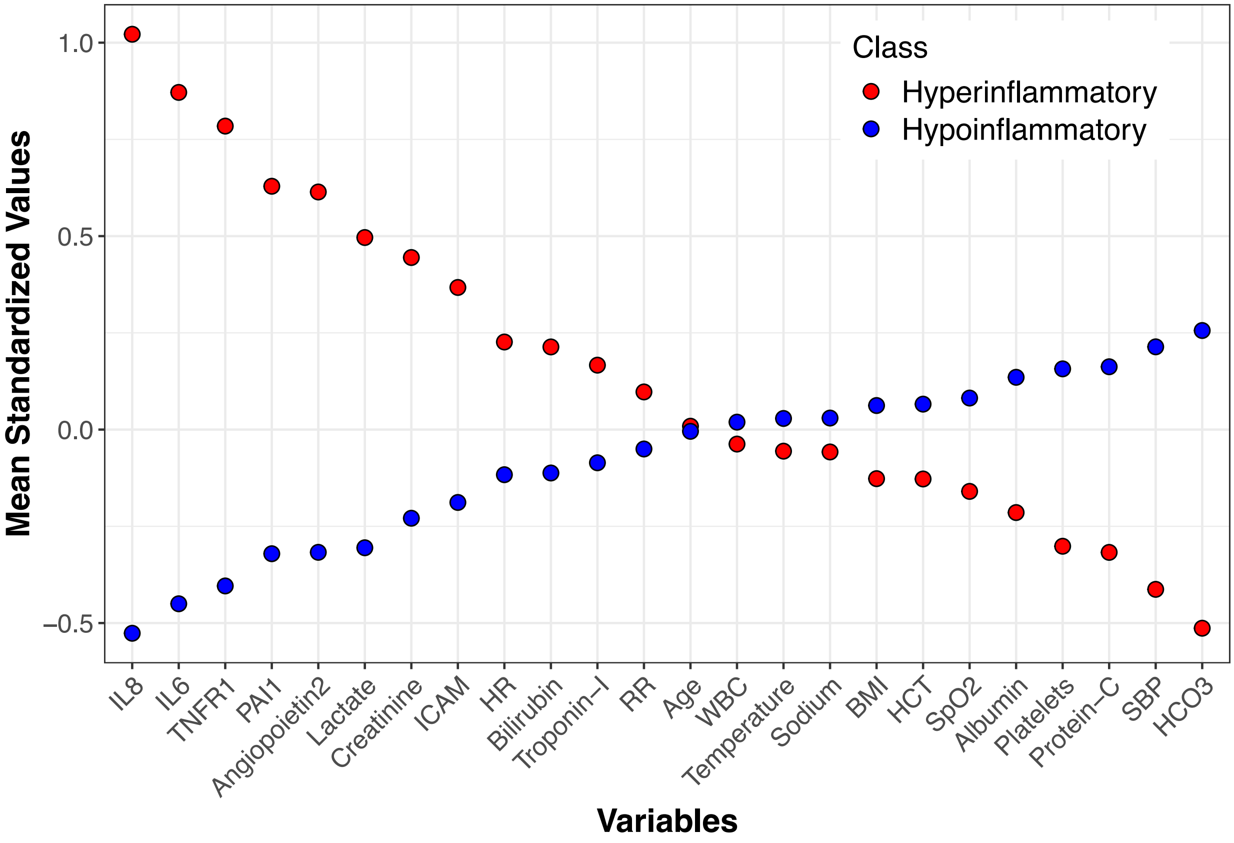

Standardized values were calculated by subtracting the mean of each variable from each observation and dividing that value by the standard deviation of each variable. The variables were arranged in descending order of standardized values in the hyperinflammatory phenotype. All biomarker concentrations are natural log transformed. BMI, body mass index; HCO3, bicarbonate; HR, heart rate; ICAM-1, intercellular adhesion molecule-1; IL, interleukin; PAI- 1, plasminogen activator inhibitor-1; RR, respiratory rate; SBP, systolic blood pressure; SpO2, peripheral O2 saturation; sTNFr1, tumor necrosis factor receptor-1; WBC, white cell count.

Availability: Age (100%), BMI (90.1%), temperature (100%), heart rate (100%), systolic blood pressure (99.1%), SpO2 (99.1%), respiratory rate (100%), WBC (100%), hematocrit (100%), platelets (99.3%), sodium (100%), HCO3 (99.6%), creatinine (100%), lactate (17.6%), albumin (52.4%), bilirubin (65.7%), IL-6 (99.3%), IL-8 (100%), TNFr1 (100%), ICAM-1 (99.3%), Protein C (99.0%), PAI-1 (99.2%), angiopoietin-2 (99.8%), Troponin-1 (100%).

e-Figure 4: Distribution of serum biomarkers by inflammatory subphenotype and probability of subphenotype assignment.

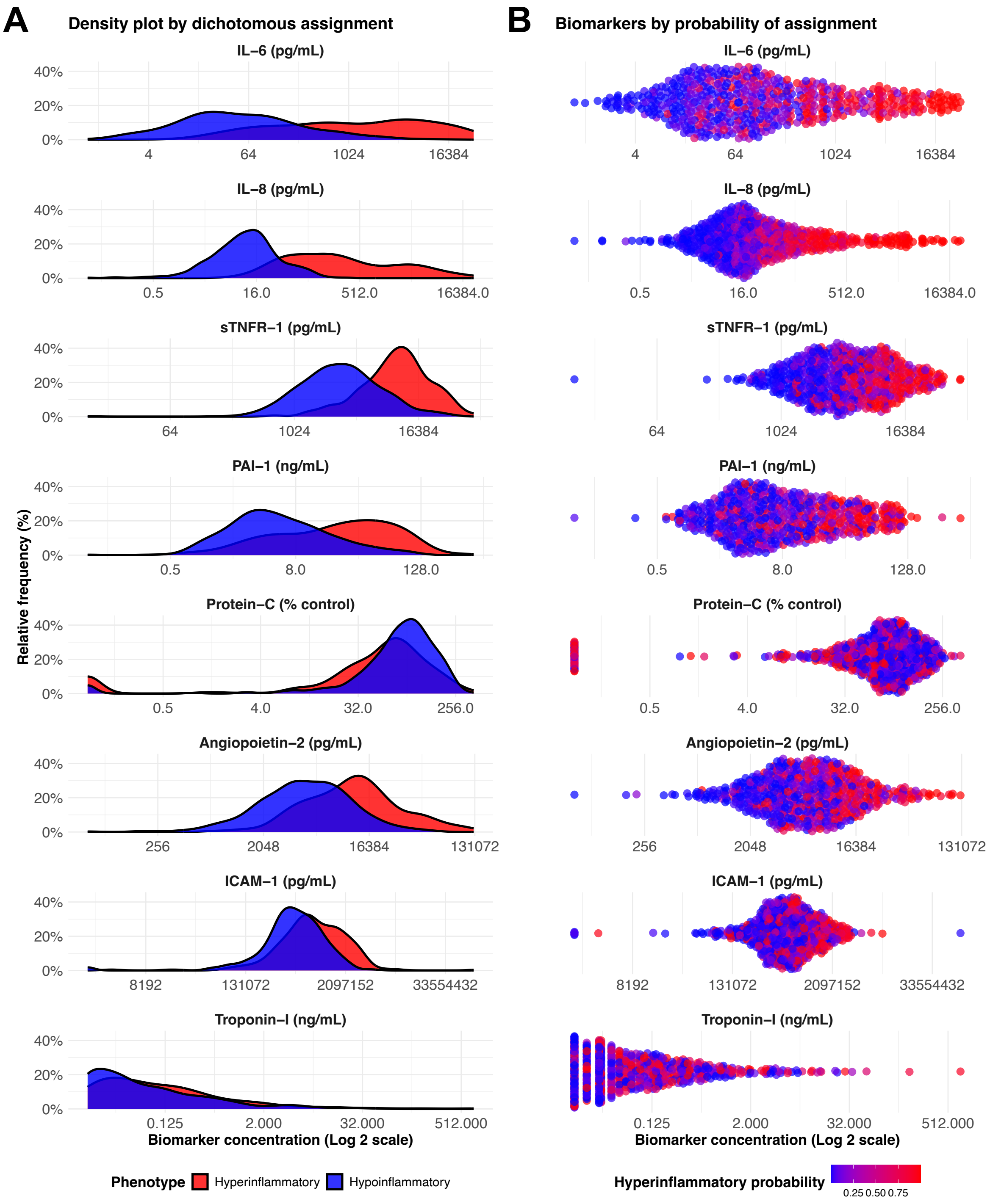

Panel A) Distribution of biomarker by assignment as hyperinflammatory (red) or hypoinflammatory (blue). **Panel B**) Distribution of biomarker concentrations by probability of assignment to hyperinflammatory. IL, interleukin; PAI- 1, plasminogen activator inhibitor-1; sTNFr1, tumor necrosis factor receptor-1

e-Figure 5: Sensitivity analysis: Crude and adjusted odds ratios for 60-day mortality associated with peak troponin-I in EARLI using classification by IL-8, sTNFr-1 and bicarbonate.

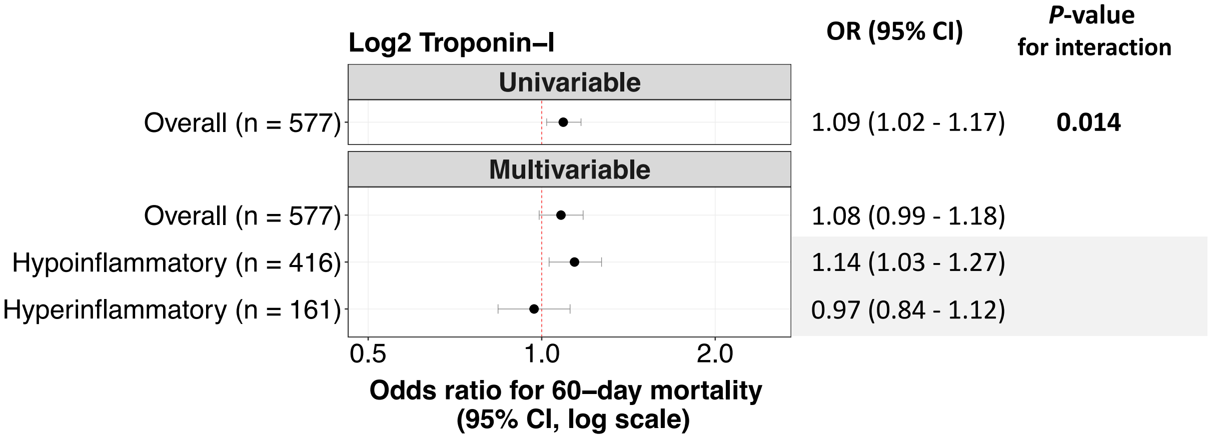

Adjusted for age, hypertension, diabetes, coronary artery disease, congestive heart failure, acute coronary syndrome, admission hematocrit, log-transformed white blood cell count, log-transformed creatinine, respiratory rate, heart rate, vasopressor on day 1, and invasive ventilation on day 1.

e-Figure 6: Sensitivity analyses: Crude and adjusted odds ratios for 60-day mortality associated with peak troponin-I in EARLI.

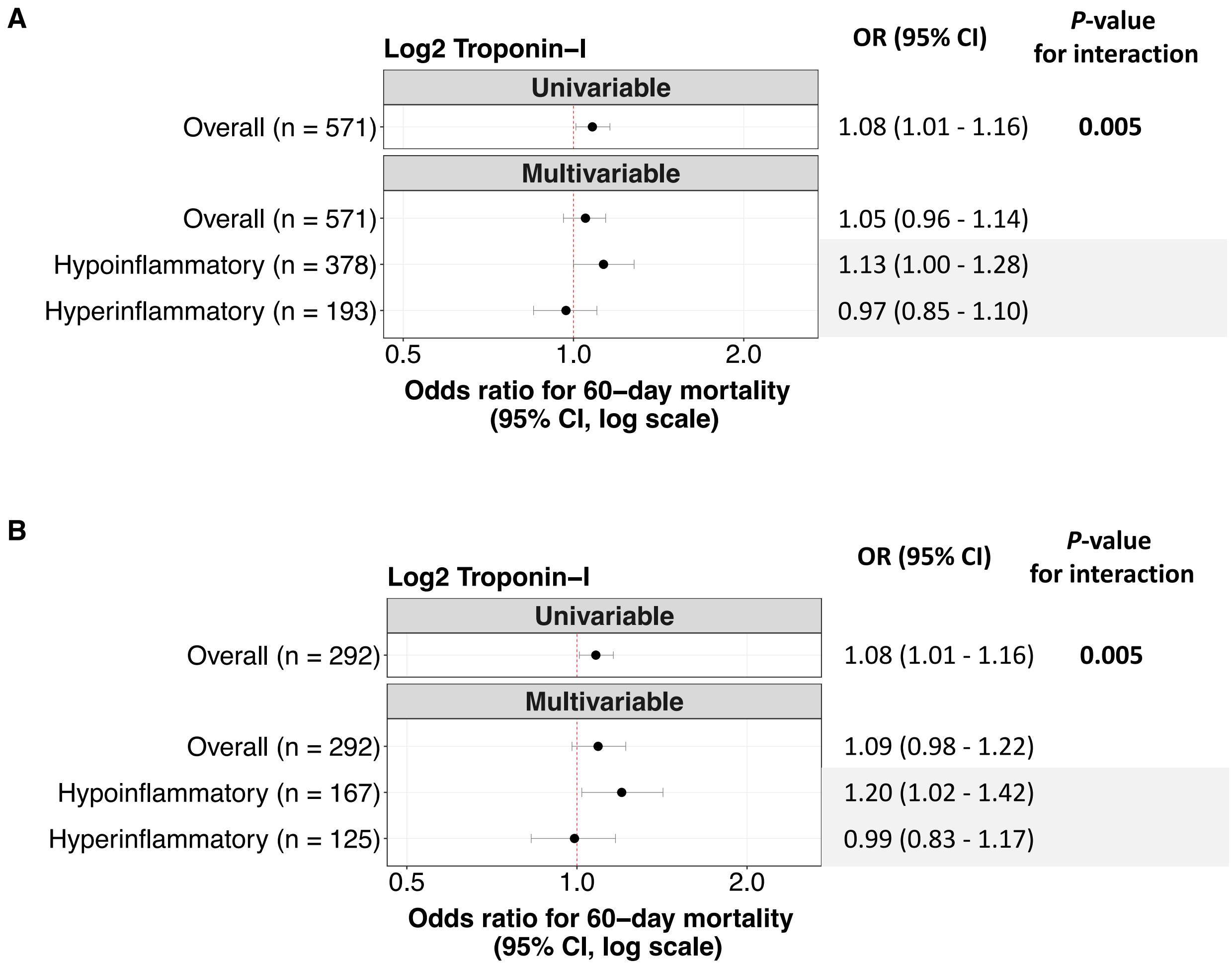

Panel A shows forest plot of univariable and multivariable analyses when including only patients with sepsis. **Panel B** shows analysis when including only patients with ARDS.

Adjusted for age, hypertension, diabetes, coronary artery disease, congestive heart failure, acute coronary syndrome, admission hematocrit, log-transformed white blood cell count, log-transformed creatinine, respiratory rate, heart rate, vasopressor on day 1, and invasive ventilation on day 1.

e-Figure 7: Subphenotype-specific analysis of longitudinal admission troponin-I by in-hospital mortality, in EARLI.

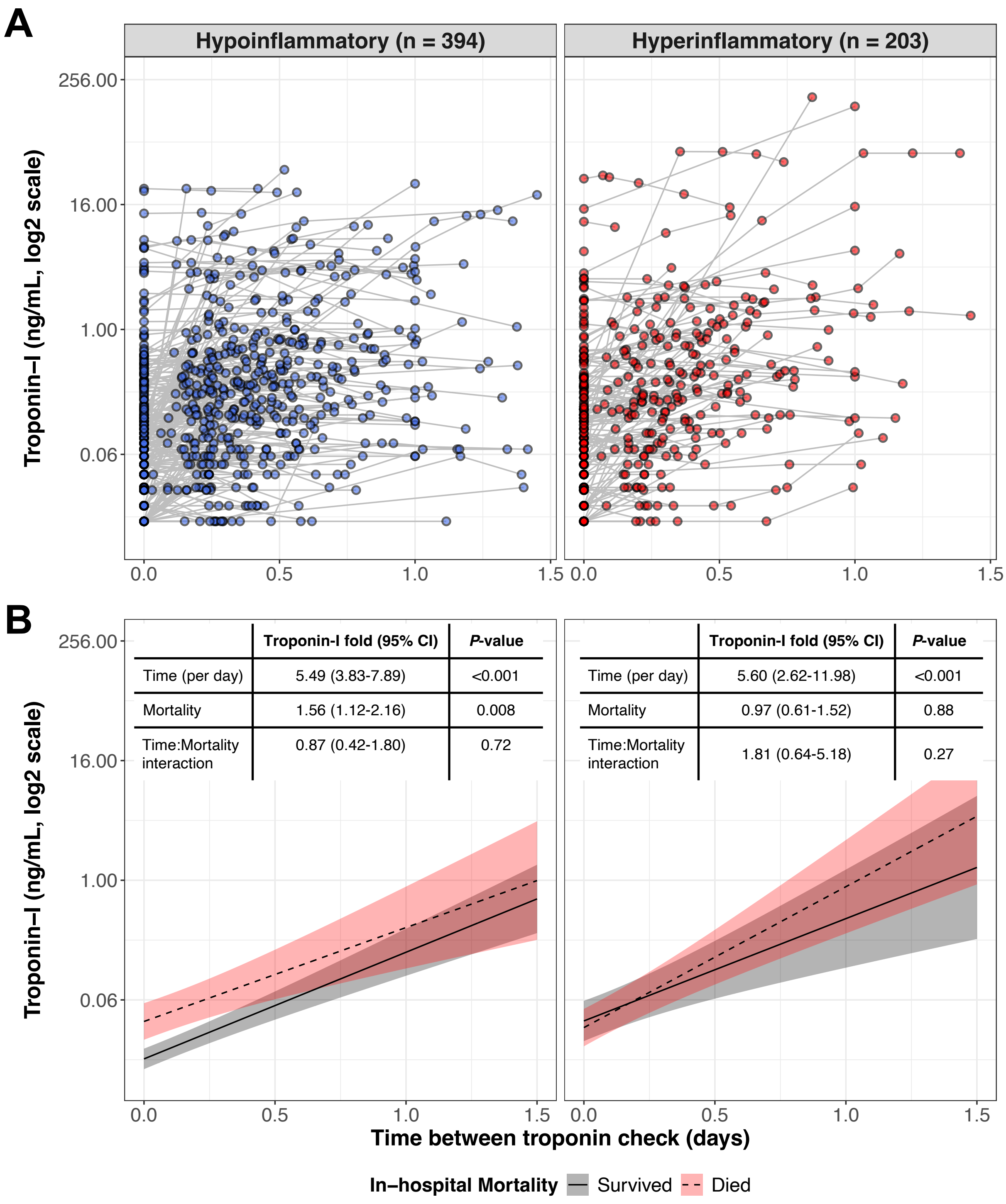

Hypoinflammatory (n = 394):

	Troponin-I fold (95% CI)	*P*-value
Time (per day)	5.49 (3.83-7.89)	<0.001
Mortality	1.56 (1.12-2.16)	0.008
Time:Mortality interaction	0.87 (0.42-1.80)	0.72

Hyperinflammatory (n = 203):

	Troponin-I fold (95% CI)	*P*-value
Time (per day)	5.60 (2.62-11.98)	<0.001
Mortality	0.97 (0.61-1.52)	0.88
Time:Mortality interaction	1.81 (0.64-5.18)	0.27

Panel A) Admission troponin-I trend by inflammatory subphenotype. **Panel B**) Multivariable linear mixed model of troponin-I concentrations by 60-day in-hospital mortality and its interaction with time, within each subphenotype. Adjusted for the same covariates as **e-Figure 2**.

e-Figure 8: Spearman correlation map of selected plasma biomarkers and Troponin-I in EARLI.

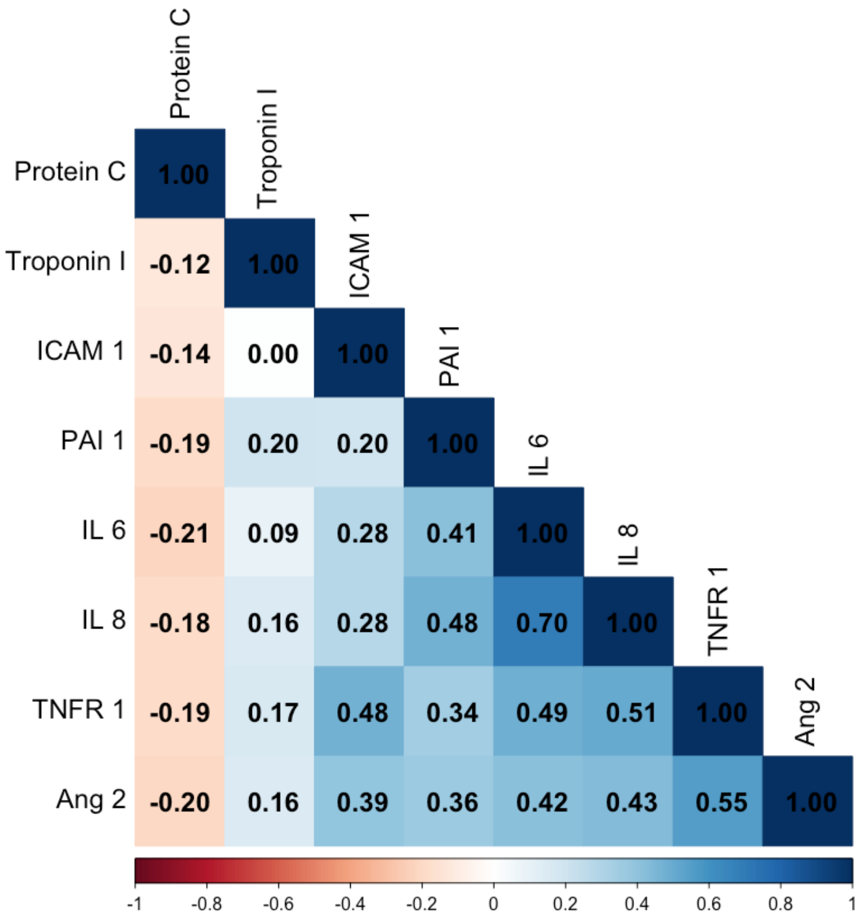

Correlation map created for patients with complete biomarker data to allow pairwise comparisons (n = 585 of 597 patients). Data presented as Spearman's rho. Red denotes negative correlation; blue denotes positive correlation.

Ang 2, angiopoietin-2; ICAM- 1, intercellular adhesion molecule-1; IL, interleukin; PAI- 1, plasminogen activator inhibitor-1; sTNFr1, tumor necrosis factor receptor-1.

e-Figure 9: Spearman correlation network of selected plasma biomarkers and peak troponin-I according to inflammatory subphenotype in EARLI.

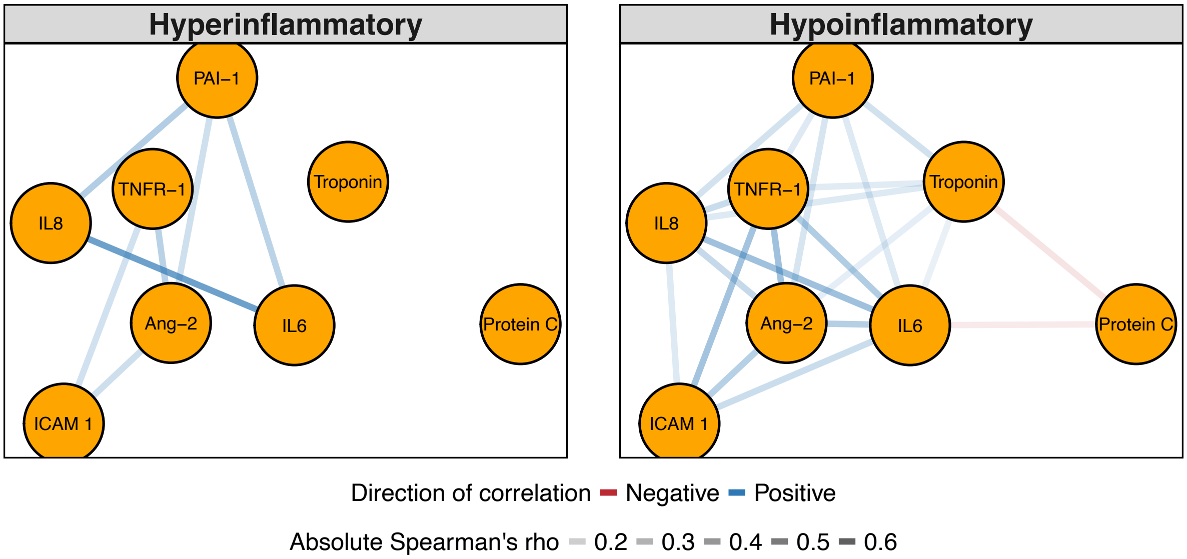

Correlation network shows Spearman's rho above 0.1 and adjusted p-values < 0.05. Hypoinflammatory phenotype n = 394; hyperinflammatory phenotype n = 203.

e-Figure 10: Crude and adjusted odds ratios for 60-day mortality associated with peak troponin-I in VALID cohort.

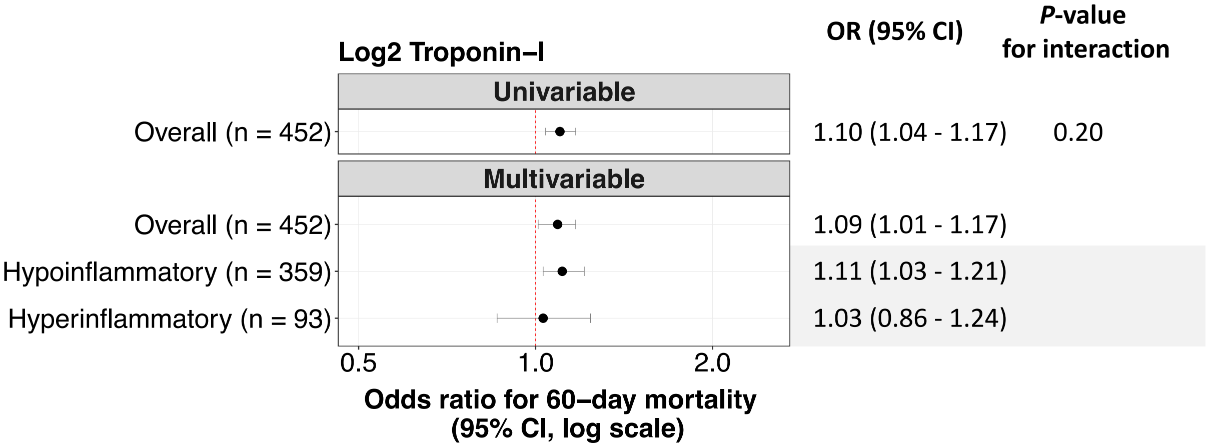

Adjusted for age, hypertension, diabetes, coronary artery disease, congestive heart failure, acute coronary syndrome, admission hematocrit, log-transformed white blood cell count, log-transformed creatinine, respiratory rate, heart rate, vasopressor on day 1, and invasive ventilation on day 1.

e-Figure 11: Sensitivity analysis: Crude and adjusted odds ratios for 60-day mortality associated with peak troponin-I after imputation of admission troponin-I values in EARLI.

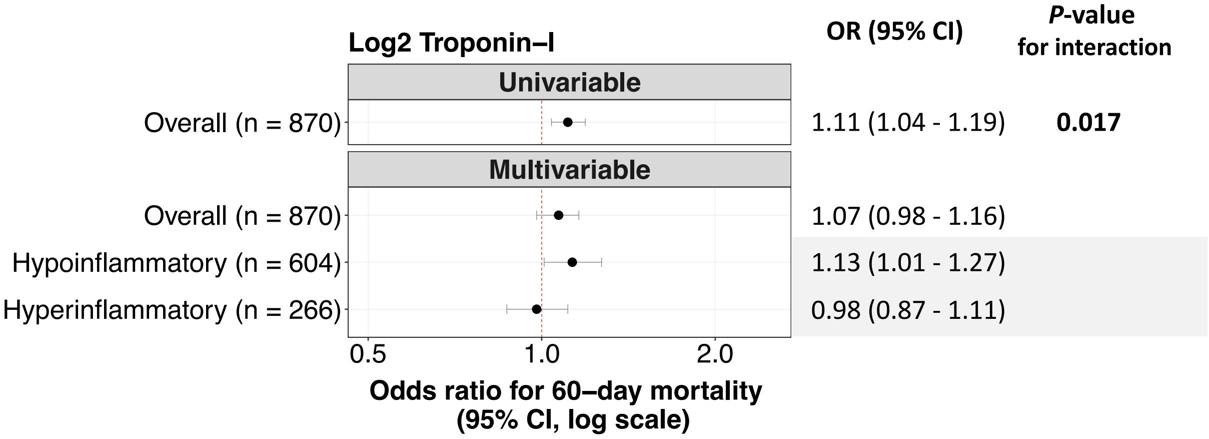

Adjusted for age, hypertension, diabetes, coronary artery disease, congestive heart failure, acute coronary syndrome, admission hematocrit, log_2-transformed white blood cell count, log-transformed creatinine, respiratory rate, heart rate, vasopressor on day 1, and invasive ventilation on day 1

e-REFERENCES

1. Knaus WA, Draper EA, Wagner DP, Zimmerman JE. APACHE II: a severity of disease classification system. *Crit Care Med*. 1985;13(10):818-829.

2. Rice TW, Wheeler AP, Bernard GR, et al. Comparison of the SpO2/FIO2 ratio and the PaO2/FIO2 ratio in patients with acute lung injury or ARDS. *Chest*. 2007;132(2):410-417. doi:10.1378/chest.07-0617.

3. Collinson PO, Saenger AK, Apple FS, IFCC C-CB. High sensitivity, contemporary and point-of-care cardiac troponin assays: educational aids developed by the IFCC Committee on Clinical Application of Cardiac Bio-Markers. *Clin Chem Lab Med*. 2019;57(5):623-632. doi:10.1515/cclm-2018-1211

4. Sinha P, Delucchi KL, Chen Y, et al. Latent class analysis-derived subphenotypes are generalisable to observational cohorts of acute respiratory distress syndrome: a prospective study. *Thorax*. 2022;77(1):13-21. doi:10.1136/thoraxjnl-2021-217158

5. Sinha P, Delucchi KL, McAuley DF, O'Kane CM, Matthay MA, Calfee CS. Development and validation of parsimonious algorithms to classify acute respiratory distress syndrome phenotypes: a secondary analysis of randomised controlled trials. *Lancet Respir Med*. 2020;8(3). doi:10.1016/S2213-2600(19)30369-8

6. Sinha P, Kerchberger VE, Willmore A, et al. Identifying molecular phenotypes in sepsis: an analysis of two prospective observational cohorts and secondary analysis of two randomised controlled trials. *Lancet Respir Med*. 2023;11(11):965-974. doi:10.1016/S2213-2600(23)00237-0
